# The WOPR Protein Ros1 Is a Master Regulator of Sporogenesis and Late Effector Gene Expression in the Maize Pathogen *Ustilago maydis*

**DOI:** 10.1371/journal.ppat.1005697

**Published:** 2016-06-22

**Authors:** Marie Tollot, Daniela Assmann, Christian Becker, Janine Altmüller, Julien Y. Dutheil, Carl-Eric Wegner, Regine Kahmann

**Affiliations:** 1 Max Planck Institute for Terrestrial Microbiology, Department of Organismic Interactions, Marburg, Germany; 2 Cologne Center for Genomics (CCG), University of Cologne, Cologne, Germany; 3 Max Planck Institute for Terrestrial Microbiology, Deparment of Biogeochemistry, Marburg, Germany; Nanjing Agricultural University, CHINA

## Abstract

The biotrophic basidiomycete fungus *Ustilago maydis* causes smut disease in maize. Hallmarks of the disease are large tumors that develop on all aerial parts of the host in which dark pigmented teliospores are formed. We have identified a member of the WOPR family of transcription factors, Ros1, as major regulator of spore formation in *U*. *maydis*. *ros1* expression is induced only late during infection and hence Ros1 is neither involved in plant colonization of dikaryotic fungal hyphae nor in plant tumor formation. However, during late stages of infection Ros1 is essential for fungal karyogamy, massive proliferation of diploid fungal cells and spore formation. Premature expression of *ros1* revealed that Ros1 counteracts the *b*-dependent filamentation program and induces morphological alterations resembling the early steps of sporogenesis. Transcriptional profiling and ChIP-seq analyses uncovered that Ros1 remodels expression of about 30% of all *U*. *maydis* genes with 40% of these being direct targets. In total the expression of 80 transcription factor genes is controlled by Ros1. Four of the upregulated transcription factor genes were deleted and two of the mutants were affected in spore development. A large number of *b*-dependent genes were differentially regulated by Ros1, suggesting substantial changes in this regulatory cascade that controls filamentation and pathogenic development. Interestingly, 128 genes encoding secreted effectors involved in the establishment of biotrophic development were downregulated by Ros1 while a set of 70 “late effectors” was upregulated. These results indicate that Ros1 is a master regulator of late development in *U*. *maydis* and show that the biotrophic interaction during sporogenesis involves a drastic shift in expression of the fungal effectome including the downregulation of effectors that are essential during early stages of infection.

## Author Summary

The fungus *Ustilago maydis* is a pathogen of maize which induces tumor formation in the infected tissue. In these tumors huge amounts of fungal spores develop. As a biotrophic pathogen, *U*. *maydis* establishes itself in the plant with the help of a large number of secreted effector proteins. Many effector proteins are important for virulence because they counteract plant defense reactions. In this manuscript we have identified and characterized Ros1, a master regulator for the late stages of *U*. *maydis* development. This transcription factor is expressed late during infection and controls nuclear fusion, hyphal aggregation and late proliferation. *ros1* mutants are still able to induce tumor formation but these are a dead end because they do not contain any spores. We show that Ros1 interferes with the early regulatory cascade controlled by a complex of two homeodomain proteins. In addition, Ros1 triggers a major switch in the effector repertoire, suggesting that different sets of effectors are needed for different stages of fungal development inside the plant.

## Introduction

The basidiomycete *Ustilago maydis* is a biotrophic pathogen colonizing maize. The resulting disease, the so-called smut disease, is characterized by the formation of large tumors on all aerial parts of the plant. In these tumors fungal hyphae proliferate profusely and eventually produce massive amounts of dark pigmented, diploid teliospores.

*U*. *maydis* is a dimorphic fungus which can grow by yeast-like budding in the absence of a host. On the leaf surface compatible haploid yeast-like cells mate and generate a dikaryon. The dikaryon switches to a filamentous form which is cell cycle arrested [1–3]. Upon perception of surface cues [4] the dikaryon differentiates infection structures and penetrates the maize epidermis. Following penetration, the cell cycle arrest is released [2], the dikaryon invades the plant tissue, proliferates with the help of clamp formation and triggers the development of large tumors [2, 5]. In the late stages of infection, after tumors are formed, the sporogenesis program is initiated. Although the chronology of events leading to teliospore formation is not yet fully understood, the first step is likely to be the fusion of the two haploid nuclei, followed by extensive mitotic divisions of the diploid hyphal cells leading to the formation of large hyphal aggregates [5, 6]. Concomitantly, a mucilaginous matrix of undefined composition and origin is formed embedding the fungal cells during the subsequent hyphal fragmentation and maturation stages. Eventually, the tumors rupture and release the diploid spores in the environment. The cycle is completed when the teliospores germinate and give rise to haploid progeny after meiosis [6]. To overcome PAMP-triggered plant defense responses, and to establish a biotrophic interaction, *U*. *maydis* secretes a large panel of effector proteins which may function in the apoplast (e.g. Pep1) or be translocated to the host cells (e.g. Cmu1, Tin2) [7–9]. Expression of the vast majority of the about 300 effectors lacking known protein domains is tied to the biotrophic stage [10]. About 25% of all effectors are arranged in gene clusters and many of these affect virulence either generally or in an organ-specific manner [10–13]. So far, the molecular basis for the virulence function of only a few *U*. *maydis* effectors (Pep1, Pit2, Cmu1, Tin2, See1) has been elucidated [8, 9, 14–16].

*U*. *maydis* pathogenic development requires fusion of haploid cells and is initiated by the *a* and *b* mating type genes. Their expression is induced by the pheromone response factor Prf1 in response to pheromone and host derived signals transmitted via a cAMP-dependent and a MAPK pathway. The *a* locus encodes a pheromone/receptor system mediating cell-cell recognition and fusion. The *b* locus encodes two homeodomain proteins which, when derived from different alleles, form the bE/bW heterodimer which acts as master regulator for the switch to filamentous growth, host tissue colonization and tumor induction [10, 17]. bE/bW induces a regulatory cascade which influences the expression of over three hundreds genes [3, 17]. The majority of these genes are regulated via the zinc-finger protein Rbf1, a direct target of bE/bW which acts as a central node in the *b*-regulatory cascade [3]. Because effector gene expression coincides with pathogenic development, the induction of most effector genes was initially considered to depend on the *b* cascade [10]. However only few effector genes are subject to direct regulation via components of this cascade. Rbf1 induction in axenic culture leads to activation of only a small subset of effector genes suggesting that plant signals and additional regulators are required for their expression [3, 17]. The membrane proteins Sho1 and Msb2 induce effector gene expression in response to surface cues prior to penetration. They act via the transcription factors Biz1 and Hdp2, two Rbf1 targets, with a specific function in appressorium development [1, 18]. Biz1 might also regulate effector gene expression in conjunction with Mzr1 in the later stages of colonization [19].

Proteins of the WOPR family constitute a novel class of fungal-specific transcriptional regulators that bind DNA via their N-terminal WOPR box. The WOPR box consists of two highly conserved domains, WOPRa and WOPRb, predicted to adopt a globular structure and which are separated by a linker region of variable length and sequence. Both WOPR domains are required for DNA binding activity [20]. Most fungal genomes contain two paralogous *WOPR* genes that phylogenetically fall into two distinct clades [21, 22]. To date, WOPR proteins have been studied exclusively in ascomycetes where they fulfill a conserved function in the control of developmental processes. Mit1p in *Saccharomyces cerevisiae* is a core regulator of invasive growth in haploid cells as well as pseudohyphal growth in diploid cells [23, 24]. Wor1, the best characterized member of the WOPR family, is the master regulator of the white-opaque phenotypic switching allowing *Candida albicans* to adapt to niches in the human host [20]. Similarly, the Ryp1 protein in *Histoplasma capsulatum* is a key regulator of the temperature-dependent mycelia-to-yeast transition critical for virulence [25].

Sge1 was the first WOPR protein studied in a plant pathogenic fungus. Sge1 supports parasitic growth of the tomato wilt pathogen *Fusarium oxysporum f*. *sp*. *lycopersici* by inducing the expression of at least four of the *SIX* effector genes [26]. WOPR proteins have later been linked to virulence in other ascomycete plant pathogens [27–31]. Several members of the WOPR family in plant pathogens also positively regulate the production of secondary metabolites potentially involved in pathogenicity [27–29]. In addition to their role in plant colonization, most WOPR proteins regulate sexual/asexual reproduction in phytopathogenic fungi [26–28, 30–32].

Here we investigate the function of a WOPR regulator in the pathogenic development of the basidiomycete pathogen *U*. *maydis*. We show that Ros1 (Regulator of sporogenesis 1) is not required for plant colonization but is essential for teliospore production occurring late during the biotrophic life cycle. *ros1* deletion strains are locked in the dikaryotic, filamentous stage of infection. They fail to undergo karyogamy, subsequent mitotic cell divisions and are unable to form the mucilaginous matrix in which teliospores are embedded. We show that Ros1 affects the expression of many genes via direct interaction with their promoter regions. Remarkably, Ros1 triggers a dramatic switch in gene expression of the vast majority of effector genes.

## Results

### Ros1 is a potential transcriptional regulator

The genome of *U*. *maydis* is predicted to encode two members of the WOPR family, *pac2* (*UMAG_15096*) and *ros1* (*UMAG_05853*). As the deletion of *pac2* (*UMAG_15096*) was previously shown to not have any effect on *U*. *maydis* virulence or reproduction [33], only *ros1* was investigated here.

A BLAST search revealed that Ros1 is conserved in other smut species belonging to the four genera of the class Ustilaginomycetes (*Ustilago*, *Sporisorium*, *Pseudozyma*, and *Melanopsichium*). Outside of the Ustilaginomycetes, conservation is restricted to the N-terminal part comprising the WOPR box (S1 Fig). In Ros1 the N-terminal WOPR box (amino acids 8 to 305) contains both WOPRa (amino acids 8 to 90) and WOPRb (amino acids 240 to 305) domains separated by a rather long linker region of 156 amino acids (S1A Fig). With the exception of Thr210, all residues critical for DNA binding in Wor1 of *C*. *albicans* are conserved in Ros1 (S1B Fig) [34]. With a length of 879 amino acids Ros1 is the longest WOPR protein described so far (S1A Fig).

Several members of the WOPR family share a conserved nuclear localization signal (NLS) (PGEKKRA) (S1 Fig). This motif is absent in Ros1 and we could not identify any other canonical NLS. However, bioinformatic tools predict Ros1 to localize in the nucleus. In addition Ros1 displays an unusually long polyglutamine stretch (46 residues, amino acids 707–752) in its C-terminal domain. Several other members of the WOPR family possess one or more glutamine-rich regions (S1A Fig) which might mediate the interaction with components of the transcriptional machinery [35].

**Fig 1:**
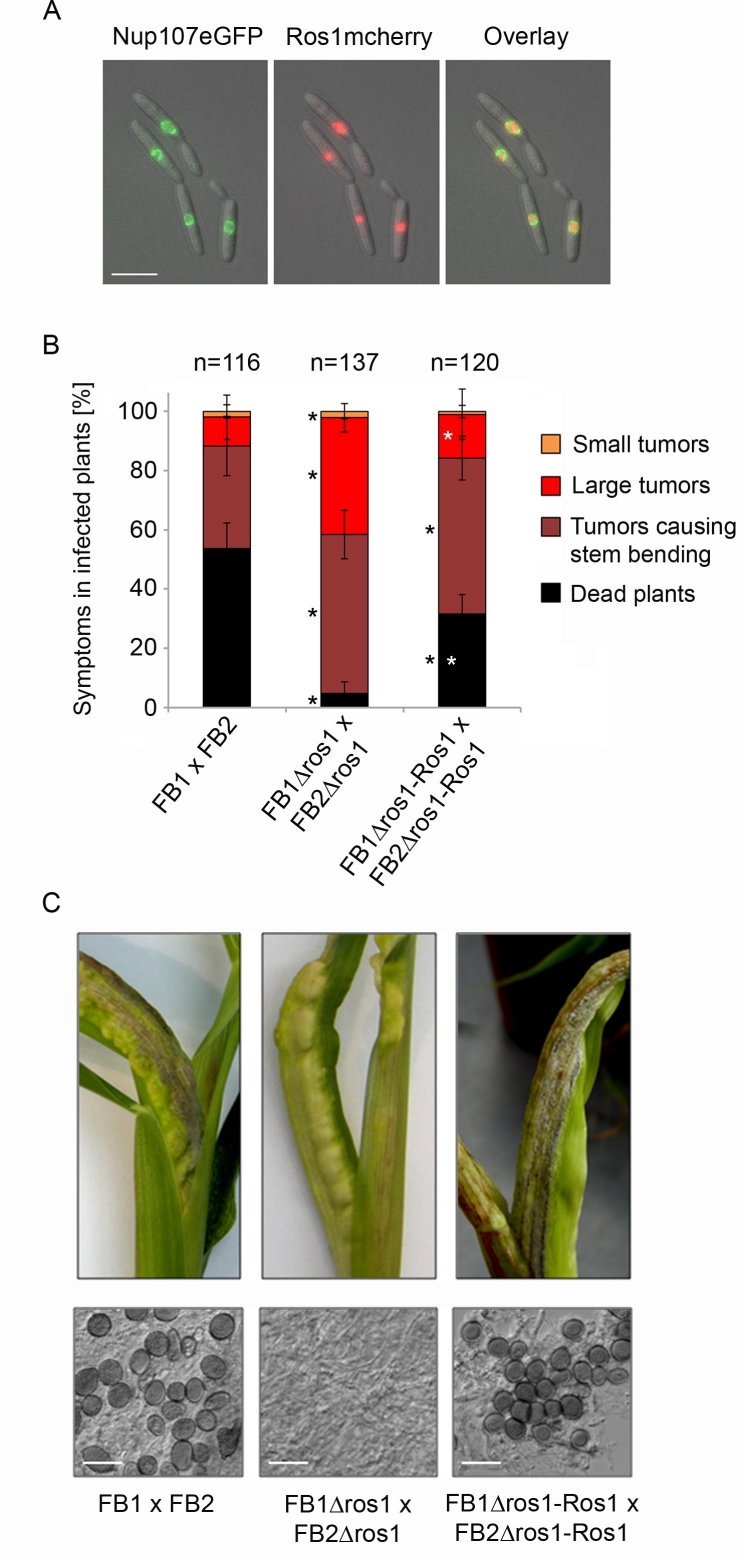
The nuclear protein Ros1 contributes to virulence and is essential for spore formation. (A) Cellular localization of Ros1. FB1_Potef_Ros1mCherry-P_nup107_Nup107eGFP expressing a Ros1mCherry fusion protein and the nuclear envelope marker Nup107eGFP were grown to mid exponential phase in YEPSL and analyzed by fluorescence microscopy. The green and red fluorescing signals corresponding to Nup107GFP and Ros1mCherry are shown in the left and middle panels, respectively. The right panel shows an overlay of both signals. The size marker corresponds to 10 μM. (B) The deletion of *ros1* attenuates virulence. Wild type strains FB1 and FB2, the *ros1* deletion strains FB1Δros1 and FB2Δros1 and the complementation strains FB1Δros1-Ros1 and FB2Δros1-Ros1 were mixed in the indicated combinations and injected into maize seedlings. Disease symptoms were scored 12 days after infection according to [10]. Colors used for disease scores are indicated on the right side. Three independent experiments were performed and the average values are expressed as a percentage of the total number of infected plants (n) given above each column. Error bars indicate standard deviation. Statistically significant differences between FB1 x FB2 and *ros1* deletion strains FB1 x FB2 and the complementation strains are indicated by black stars. Statistically significant differences between *ros1* deletion strains and the complementation strains are indicated by white stars (one-way ANOVA applying the Tukey-Kramer test[36]) (C) Representative tumors formed after infection of maize seedlings with the indicated combinations of strains are shown 12 days after infection (top panel). In the examples chosen, stem-bending is induced. The *ros1* deletion strains induce tumors, but these lack dark coloration typical for tumors containing mature spores. Lower panel: dispersed tumor tissue of the indicated strains was analyzed by light microscopy. Mature teliospores are absent from tumors induced by the *ros1* deletion strains. The size bar corresponds to 20 μM.

To study the localization of Ros1, a haploid FB1 strain was generated which constitutively expresses a C-terminal mCherry fusion of Ros1 together with the nuclear envelope marker Nup107eGFP. The red signal from Ros1mCherry was detected in the nucleus, surrounded by the green fluorescence of the nuclear envelope, demonstrating that Ros1 is targeted to the nucleus (Fig 1A). The nuclear localization and the presence of the long polyQ stretch are consistent with a potential function of Ros1 as transcriptional regulator.

### Ros1 regulates teliospore formation

To study its function we deleted *ros1* in the two compatible haploid strains FB1 and FB2. FB1Δros1 and FB2Δros1 could successfully mate and produce dikaryotic filaments on charcoal containing medium (S2 Fig). When injected into maize seedlings, nearly all plants infected with the *ros1* deletion strains developed tumors. However, compared to the FB1 x FB2 mixture, *ros1* mutants exhibited reduced virulence (Fig 1B). In particular, only 4.8% of the plants infected with the *ros1* mutant mixture were dead after 12 days compared to 53.7% for the FB1 x FB2 infection. Remarkably, dark pigmented teliospores were absent in the tumors induced by the *ros1* deletion strains (Fig 1C) and softening of the tumor tissue which usually becomes evident when teliospores accumulate in wild type infections did not occur. Spore formation could be restored by reintroducing *ros1* in single copy in the *ip* locus of FB1Δros1 and FB2Δros1 strains (Fig 1B). Because constructs containing a promoter region of 1 kb did not complement, the entire 7.6 kb region separating *ros1* from the upstream gene UMAG_05850 was included in the complementation construct. While the complementation strains had regained the ability to produce spores (Fig 1C), virulence was only partially complemented (Fig 1B). We speculate that this reflects a position effect resulting from integrating the construct into the *ip* locus. Alternatively, the expression of *ros1* might partially depend on distal regulatory elements that are missing in the complementation construct.

### Ros1 triggers the developmental switch towards spore formation

**Fig 2:**
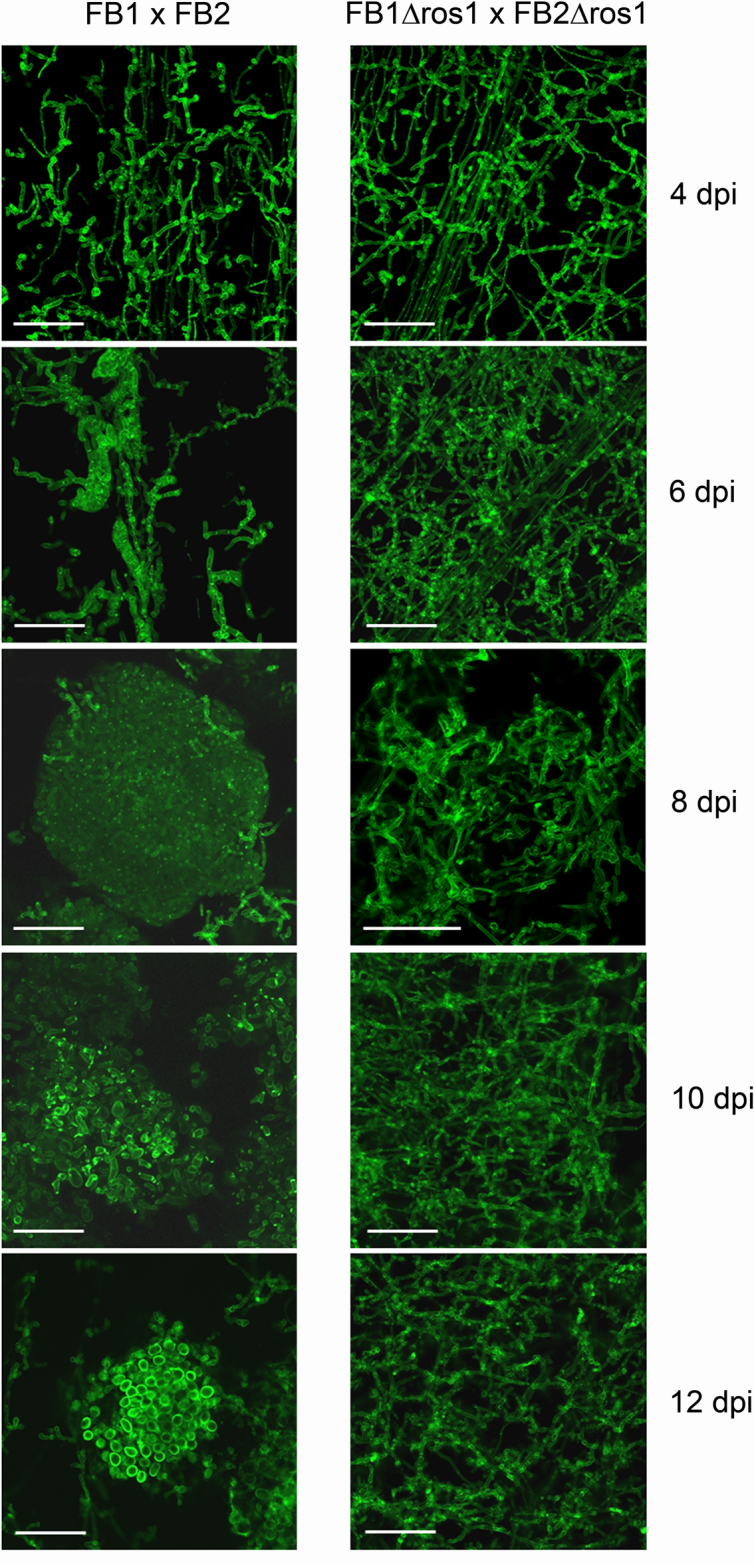
*ros1* deletion strains fail to aggregate and form spores. Tumor samples from maize seedlings infected with the wild type strains FB1 x FB2 and the corresponding *ros1* deletion strains were collected at 4, 6, 8, 10, 12 dpi, stained with wheat germ agglutinin-Alexa Fluor 488 and analyzed by laser scanning confocal microscopy (bar = 50 μm). In the FB1 x FB2 infection (left column), spore formation starts at 6 dpi with the formation of hyphal aggregates which expand (8 dpi), begin to fragment (10 dpi) and develop into teliospores (12 dpi). In comparison, the *ros1* deletion strains are locked in the filamentous state, do not aggregate and do not develop teliospores (right column). The size bar corresponds to 50 μM.

To determine at which stage of development *ros1* deletion strains are affected, we stained fungal hyphae with wheat germ agglutinin-Alexa Fluor 488 and followed the sequence of events leading to the formation of mature teliospores in wild-type infections by confocal microscopy (Fig 2, left panel). Until 4 days after infection, growth of the *ros1* deletion strains was indistinguishable from the growth of wild type strains. In both cases uniform spreading of fungal hyphae within the leaf was observed (Fig 2). After 4 days wild type hyphae started to form aggregates which became visible at 6 dpi, indicating that the mucilaginous matrix in which the cells are embedded during spore formation [37] was produced (Fig 2). Between 6 and 8 dpi the hyphal aggregates continued to expand reaching diameters of up to 250 μm at 8 dpi. Around 10 dpi, hyphae in these aggregates underwent fragmentation and individual cells entered the spore maturation process. Finally, at 12 dpi groups of mature teliospores with their characteristic ornamentation became clearly visible. This sequence of events parallels what has been described [6]. By contrast, in plants infected by *ros1* deletion strains neither hyphal aggregates nor fragmented hyphae could ever be observed (Fig 2, right panel). Thus, in the absence of *ros1*, *U*. *maydis* development was locked in the filamentous stage.

**Fig 3:**
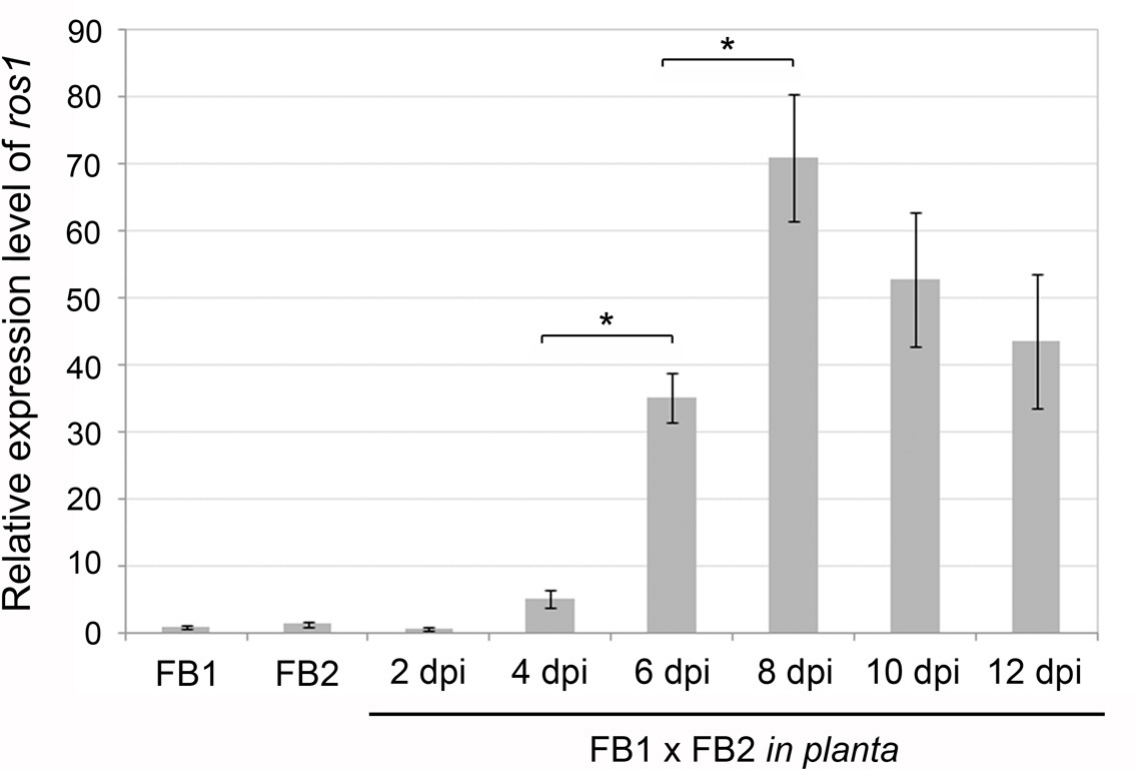
*ros1* is upregulated late after infection. qRT-PCR analysis of *ros1* expression in FB1 and FB2 grown in YEPSL or during plant infection (FB1 x FB2). Axenic culture samples were collected at OD_600_ = 1.0. Infected plant samples were collected at the time-points indicated below. qRT-PCR analysis was performed using the constitutively expressed *ppi* gene (*UMAG_03726*) for normalization. Relative expression was determined using the ΔΔCt method. Values shown are means of three biological replicates. Bars indicate the standard deviation between biological replicates. Asterisks indicate significant differences (paired *t*-test, *p* ≤ 0.05).

To see how this failure to aggregate was linked to expression of the *ros1* gene, the expression pattern of *ros1* in haploid strains in axenic culture and of FB1 x FB2 mixtures during plant infection was analyzed using quantitative RT-PCR (Fig 3). *ros1* was expressed at a very low basal level in axenic culture and during the early steps of maize infection. Expression was then upregulated 35-fold at 6 dpi when sporogenesis was initiated and reached a maximum 70-fold induction at 8 dpi as hyphal aggregates expanded. Between 8 and 12 dpi, *ros1* transcript abundance slowly decreased, concomitantly with the accumulation of mature teliospores (Fig 3).

### Ros1 controls crucial events during spore development

**Fig 4:**
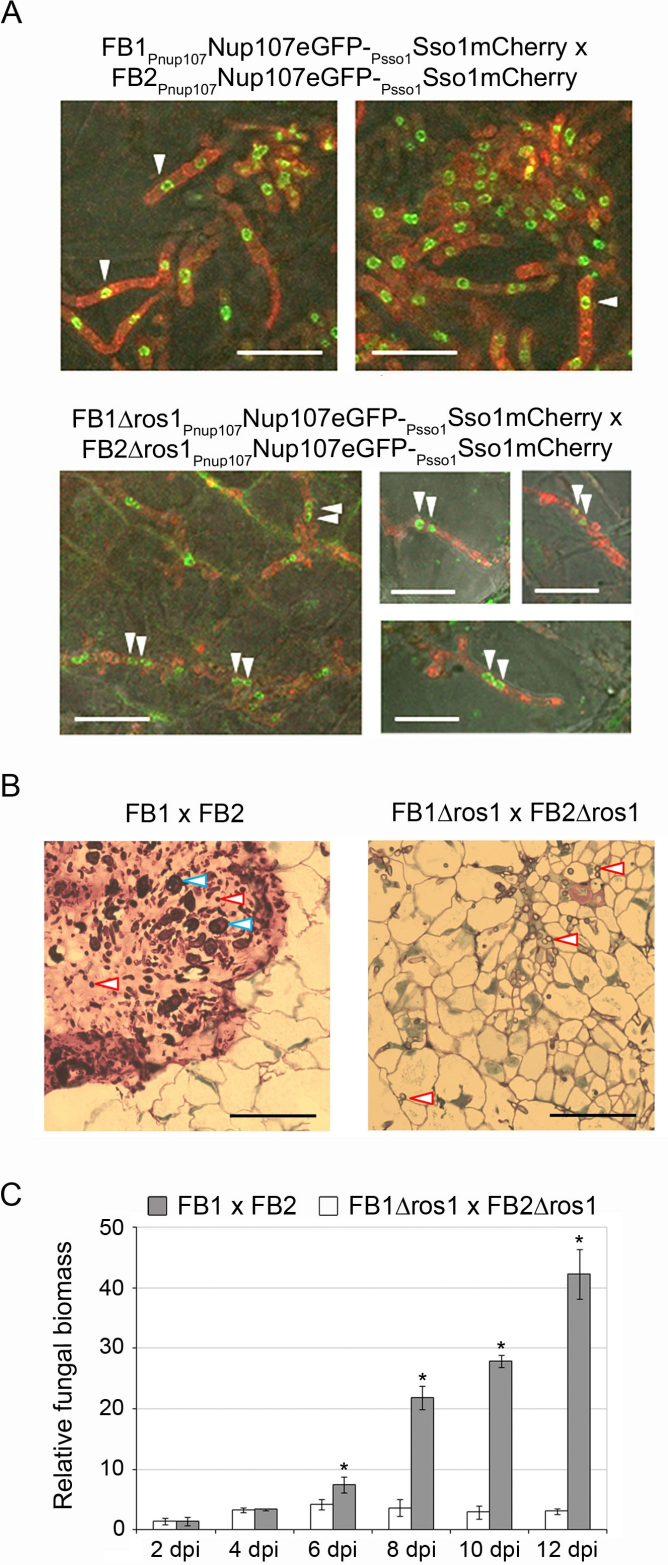
Ros1 is required for karyogamy, matrix formation and biomass increase. (A) Visualization of fungal nuclei. Nuclear status of biotrophic hyphae was assessed by imaging fungal nuclei inside hyphae in tumor tissue. Maize seedlings were infected with a combination of FB1_Pnup107_Nup107eGFP-_Psso1_Sso1mCherry x FB2_Pnup107_Nup107eGFP-_Psso1_Sso1mCherry or FB1Δros1_Pnup107_Nup107eGFP-_Psso1_Sso1mCherry x FB2Δros1_Pnup107_Nup107eGFP-_Psso1_Sso1mCherry expressing both the nuclear envelope marker Nup107eGFP (green fluorescence) and the membrane marker Sso1mCherry (red fluorescence). Tumor samples were collected between 6 and 8 dpi and analyzed by confocal microscopy. Only one nucleus is visible in hyphae of FB1_Pnup107_Nup107eGFP-_Psso1_Sso1mCherry x FB2_Pnup107_Nup107eGFP-_Psso1_Sso1mCherry (upper panel) while FB1Δros1_Pnup107_Nup107eGFP-_Psso1_Sso1mCherry x FB2Δros1_Pnup107_Nup107eGFP-_Psso1_Sso1mCherry hyphae are dikaryotic (lower panel). White arrows indicate the position of nuclei. Bar = 10 μm. (B) Staining of the mucilaginous matrix. Leaf tumors from maize seedlings infected with the indicated strains were collected at 10 dpi. Samples were fixed and embedded in Epoxy resin. 1–2 μm thick sections were generated and stained with methylene blue-azure II-basic fuchsin. The mucilaginous matrix appears in pink in the FB1 x FB2 infection while no matrix is visible in the tissue infected by FB1Δros1 x FB2Δros1. Blue arrowheads indicate maturing spores, red arrowheads indicate fungal hyphae. Bar = 50 μm. (C) Fungal biomass increase during plant infection. Plants were infected with the indicated strains and samples were collected at 2, 4, 6, 8, 10, and 12 dpi. Relative fungal biomass (grey columns FB1 x FB2; white columns FB1Δros1 x FB2Δros1) was determined by qRT-PCR using *U*. *maydis*-specific and maize-specific primers. Columns give mean ratios of fungal DNA to plant DNA from three independent experiments. The ratio in FB1 x FB1 infected plants at 2 dpi was set to 1.0. Error bars indicate standard deviation. Asterisks indicate significant differences (*t*-test, *p* ≤ 0.05).

To evaluate in more detail the contribution of Ros1 to the regulation of spore development, we analyzed karyogamy and matrix formation in the *ros1* mutant. To be able to visualize nuclei, the nuclear envelope marker Nup107eGFP as well as the plasma membrane marker Sso1mCherry were introduced into FB1, FB2 and the corresponding *ros1* deletion strains. Infected plant tissues were then analyzed by confocal microscopy between 6 and 8 dpi. At this stage wild type filaments contained only one nucleus while pairs of nuclei were visible in filaments of the *ros1* deletion strains (Fig 4A), indicating that karyogamy had not occurred (even at later time points). In addition, the mucilaginous matrix observed in tumors induced by wild type strains was absent in tumors induced by the *ros1* deletion strains (Fig 4B).

Teliospore differentiation takes place in large hyphal aggregates [5, 38]. As such aggregates were absent in tissue infected by *ros1* mutant strains, we also studied the accumulation of fungal biomass in plants infected with wild type and *ros1* deletion strains by quantitative RT-PCR (Fig 4C). In line with the microscopic data (Fig 2), until 4 dpi wild type strains as well as *ros1* mutants showed a comparable small increase in fungal biomass (Fig 4C), likely reflecting coordinated mitotic divisions of the dikaryon with the help of clamp connections [2, 3]. However, while in wild type infected tissue the ratio *U*. *maydis*/plant biomass increased dramatically (7.5 to 42.3) between 6 dpi and 12 dpi, the ratio remained at the 4 dpi level in plants infected with the *ros1* deletion strains (Fig 4C). These data show that Ros1 affects karyogamy as well as matrix formation and is needed for massive late proliferation in the infected tissue.

### Ros1 inhibits *b*-dependent regulation

**Fig 5:**
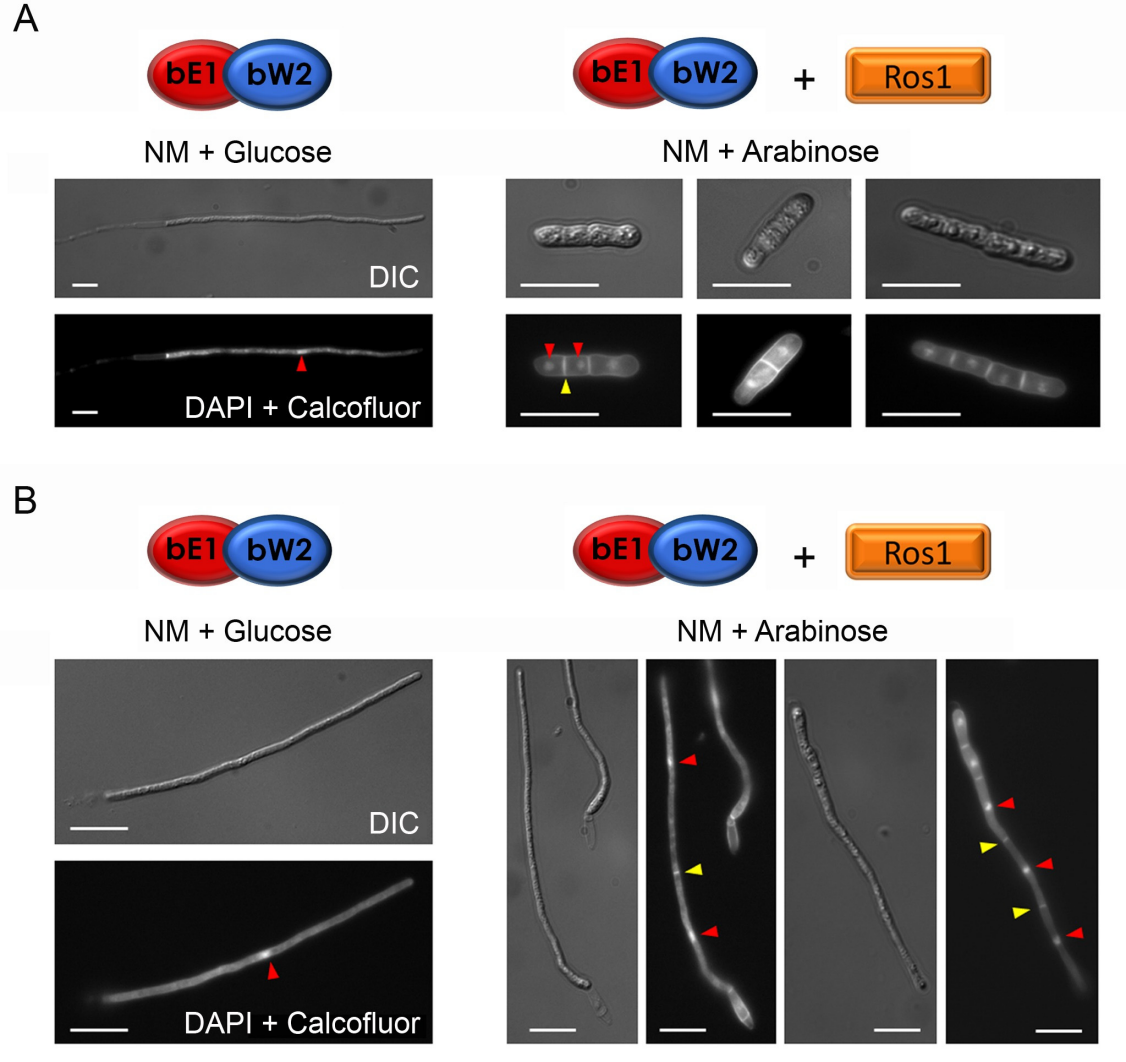
*ros1* overexpression inhibits *b-*induced filamentation and triggers septation and nuclear divisions. (A) Strain AB33_Pcrg1_Ros1 expressing *bE1* and *bW2* from the nitrate-inducible *nar1* promoter and *ros1* from the arabinose-inducible *crg1* promoter was grown in CM supplemented with glucose to an OD_600_ of 0.6 and then shifted to NM medium containing either glucose (left panel) or arabinose (right panel). In NM + glucose only the *b* genes are induced whereas in NM + arabinose expression of the *b* genes and *ros1* is induced. Samples were collected 6 h after induction. Nuclei and cell walls were stained with DAPI and calcofluor, respectively, and the morphology of the cells was assessed by DIC and fluorescence microscopy. In the presence of nitrate and glucose a functional bE1/bW2 heterodimer is produced and cells switch to filaments. In NM + arabinose, *bE1*, *bW2* and *ros1* are expressed simultaneously. In this case, cells fail to form filaments and become swollen and septated with one nucleus in each section. Bar = 10 μM. (B) AB33_Pcrg1_Ros1 was pre-incubated for 6 h in NM + glucose to allow cells to filament and then transferred to either NM + glucose (left panel) or NM + arabinose (right panel) for another 6 h. In NM + glucose the cells remained filamentous and contained one nucleus (red arrow). In NM+ arabinose, when *ros1* is induced, filaments became septated (yellow arrows) and each section contained one nucleus (red arrows). Nuclei and cell walls were stained as in A. Bar = 10 μm.

To further characterize the function of Ros1, we studied the effect of expressing *ros1* prematurely. To mimic early pathogenic development of *U*. *maydis*, we used strain AB33 in which filamentous growth and cell cycle arrest can be triggered in liquid culture via nitrate-inducible expression of *bE1* and *bW2* homeodomain genes [39, 40]. Ros1 was placed under the control of the arabinose-inducible *crg1* promoter [41]. In nitrate minimal medium supplemented with glucose (NM + Glucose), the *b* genes were expressed and cells switched from budding to filamentous growth (Fig 5A). However, when *ros1* was induced simultaneously (NM + arabinose), cells failed to filament, increased their diameter and formed septa (Fig 5A). DAPI staining revealed that each section contained one nucleus, indicating that mitotic divisions had resumed. When filamentation was induced prior to *ros1* expression, the resulting filaments stopped elongating and became septated with one nucleus per segment (Fig 5B). This shows that Ros1 counteracts the activity of the bE/bW heterodimer, inhibits filamentation and triggers mitotic divisions.

**Fig 6:**
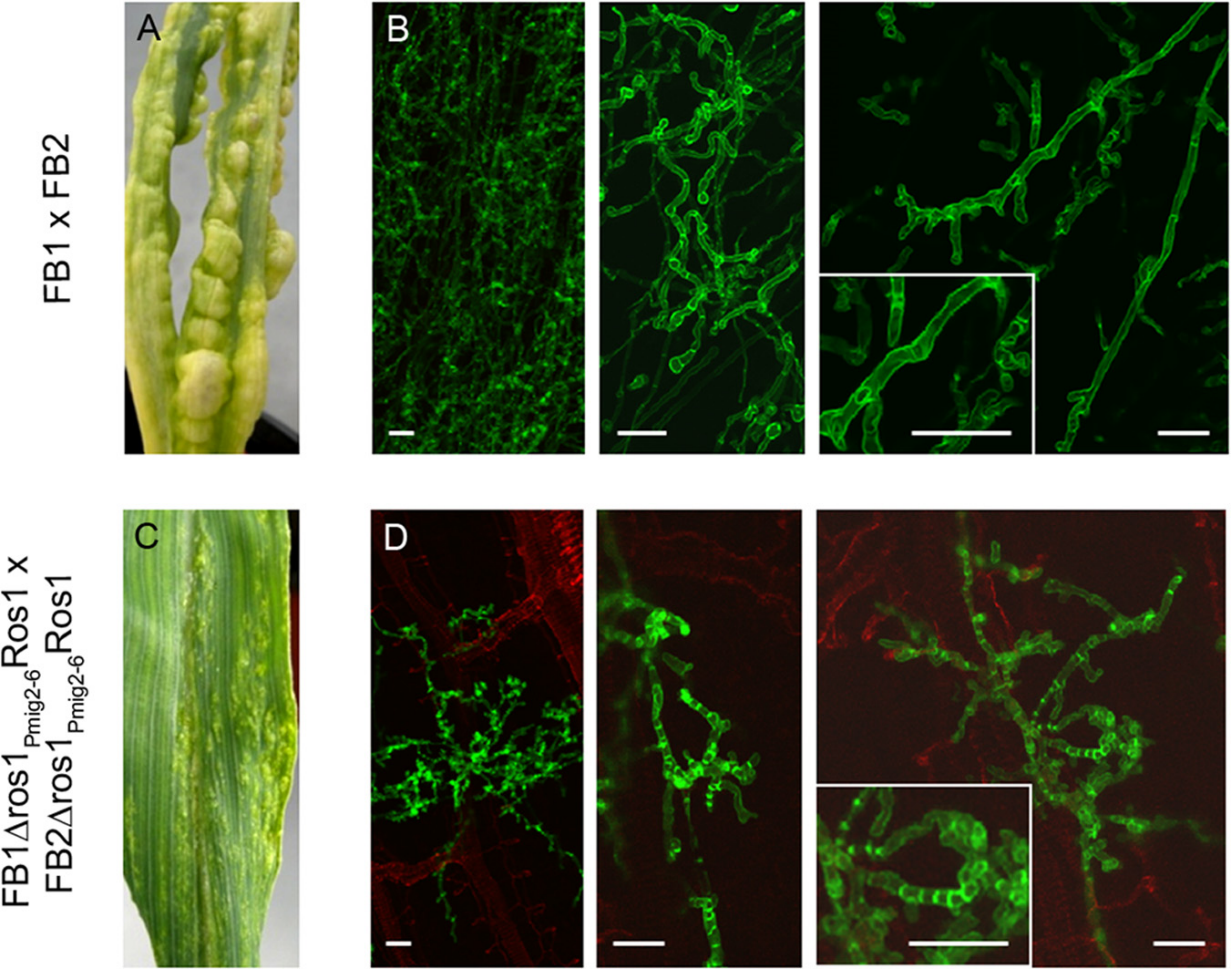
Premature expression of *ros1* triggers an early arrest of filamentous growth and induces septation. Maize seedlings were infected with a combination of FB1 x FB2 (A, B) or FB1Δros1_Pmig2-6_Ros1 x FB2Δros1_Pmig2-6_Ros1 strains expressing *ros1* from the *mig2-6* promoter which is induced two days after colonization (C, D). A and C show macroscopic symptoms on leaves at 8 dpi. Only very small tumors are visible on plants infected with FB1Δros1_Pmig2-6_Ros1 x FB2Δros1_Pmig2-6_Ros1. B and D panels show confocal microscopy pictures obtained from 3 dpi plant samples. The fungal cell wall was stained with wheat germ agglutinin-Alexa Fluor 488 (green fluorescence) and the plant cell wall was stained with propidium iodide (red fluorescence). Growth of the strains expressing *ros1* prematurely is restricted to a small area surrounding the penetration site and hyphae exhibit an altered morphology. The inserts depict enlargements of biotrophic hyphae displaying increased septation when Ros1 is prematurely expressed. Bar = 10 μm.

To study the effect of premature *ros1* expression during plant colonization, we generated compatible *ros1* deletion strains expressing *ros1* under the control of the *mig2-6* promoter. *mig2-6* is an effector gene whose expression is strongly upregulated shortly after penetration [42]. Compared to wild type infections and infections with *ros1* deletion strains (Fig 6A) the strains expressing *ros1* prematurely caused severely attenuated disease symptoms ranging from chlorosis to very small tumors (Fig 6C). These strains penetrated the plant surface but invasion of the plant tissue rapidly stopped and hyphae with an abnormally high number of septa developed (compare Fig 6B and 6D). These results show that the timing of *ros1* expression is critical for biotrophic development and tumor induction of *U*. *maydis*.

### Identification of Ros1 target genes

To identify genes regulated by Ros1, we combined two approaches: RNA sequencing to evaluate the global effect of Ros1 on gene expression and ChIP sequencing to identify which of the differentially regulated genes are direct targets. Both experiments were carried out on samples collected at 8 dpi when the *ros1* expression level is maximal.

For the transcriptomic analysis, we compared maize tumor tissue infected by strains FB1 x FB2 and the corresponding *ros1* deletion strains. RNA-seq data showed that 2005 genes were differentially regulated (fold change (FC) ≥ 1,5; *p*-value < 0.01). Of these genes, 1091 were expressed at lower levels in the wild type strains compared to *ros1* mutants and 914 were higher expressed in wild type strains compared to *ros1* mutant strains (S1 Table). This shows that about 30% of the 6766 protein-encoding *U*. *maydis* genes are differentially regulated by Ros1. RNAseq results were confirmed by qRT-PCR for 15 genes encoding two glycoside hydrolases (*UMAG_05550*, *UMAG_04503*), a trehalase (*UMAG_02212*), a cyclopropane fatty acid synthase (*UMAG_01070*), a polyketide synthase (*pks1* [43]), transcription factors (*UMAG_04101*, *biz1* [1], *rbf1* [3], *fox1* [44], *UMAG_02775*) and secreted effectors (*mig2-3* [45], *UMAG_04096*, *dik1* [46], *UMAG_02473* [10], *UMAG_03046*) (S3 Fig).

**Fig 7:**
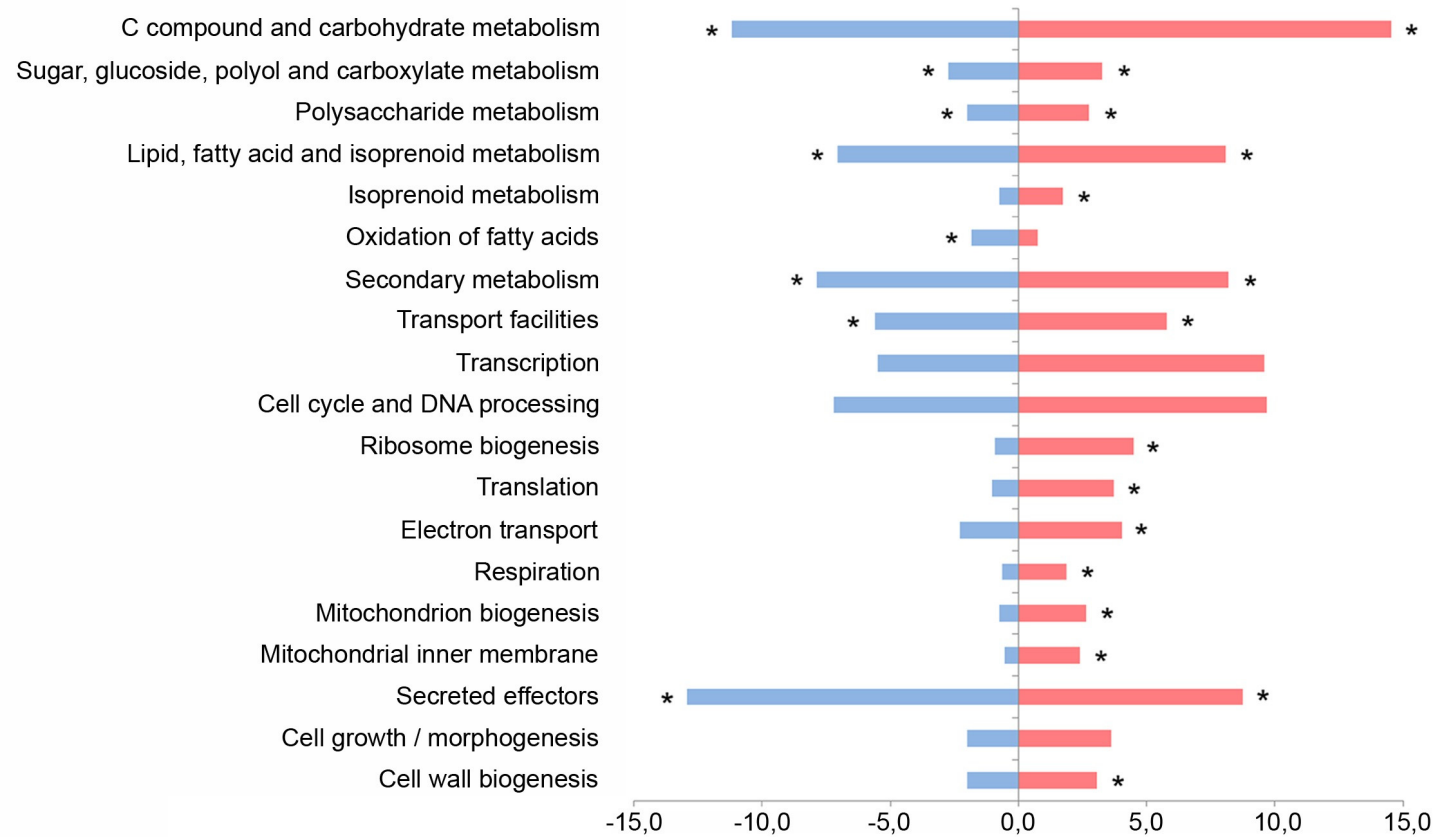
Cellular processes regulated by Ros1. Functional enrichment analysis of the differentially regulated genes identified by RNA-seq in FB1 x FB2 compared to FB1Δros1 x FB2Δros1 strains 8 days after infection of maize seedlings. Analysis was performed using the FunRich program and FunCat annotations available on the MIPS *Ustilago maydis* database (http://www.helmholtz-muenchen.de). The category “secreted effectors” was added and refers to all genes encoding secreted proteins without annotated functional domain. The chart shows the proportion of genes belonging to selected functional categories in the up-regulated (red) and down-regulated (blue) gene sets. Overrepresented categories in each up and downregulated gene sets compared to the global gene distribution are indicated with asterisks (*p* ≤ 0.05).

**Fig 8:**
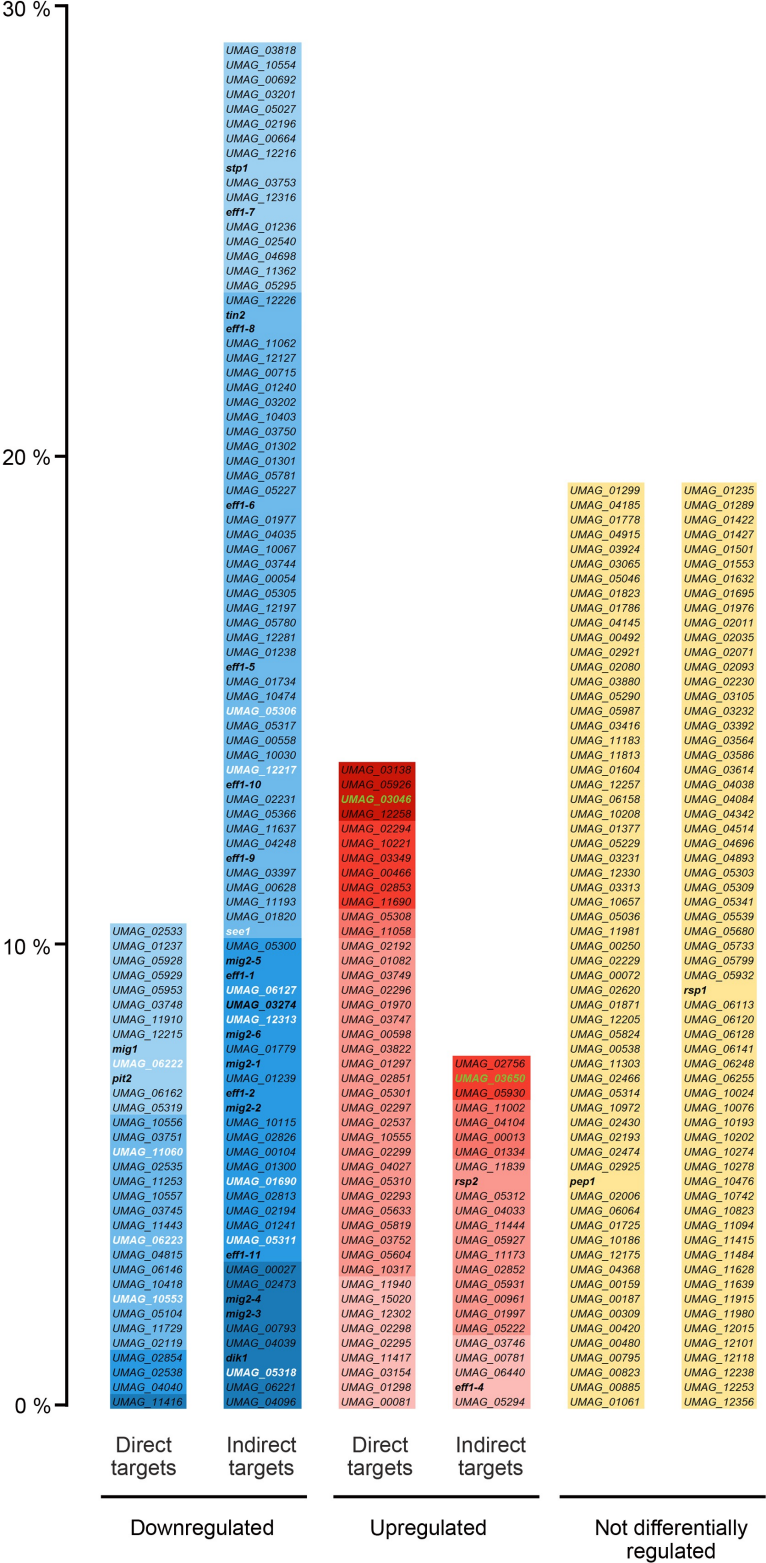
Ros1 alters the expression of the effector repertoire late during infection. All genes encoding secreted effectors without predicted functional domain are listed and their up regulation or downregulation by Ros1 is indicated. Based on the RNA-seq data comparing gene expression in FB1 x FB2 and the corresponding *ros1* deletion strains 8 days after infection of maize seedlings and the ChIP-seq data from FB1Δros1-Ros1HA x FB2Δros1-Ros1HA at the same time point, effector genes were sorted in 5 different categories presented as histograms: directly downregulated, indirectly downregulated, directly upregulated, indirectly upregulated and not differentially regulated by Ros1. The percentage (Y axis) represented by each category is calculated relative to the total number of effector genes without functional domains (320 genes based on [47] and secretion prediction by SignalP 4.1 [48]). Colors refer to the fold change of transcripts in wild type compared to *ros1* deletion strains in the RNA-seq analysis; the darker the color the higher the fold change. Downregulated effector genes are indicated in blue, upregulated effector genes are indicated in red, effector genes not regulated by Ros1 are indicated in yellow. All genes names or identification numbers are listed; genes which have been characterized or mentioned in previous publications are indicated in bold characters. Leaf-specific effector genes without functional domains [13] showing virulence defects after seedling infection are shown in white, tassel-specific effector genes without functional domains [13] are shown in green. *cmu1* [8] and three other differentially regulated effector genes (*UMAG_03615* [10], *UMAG_11763*, *UMAG_01130* [13]) are not included in this figure as they contain a functional domain.

The deletion of *ros1* mostly affects metabolic processes and cellular transport (Fig 7). Functional categories “C compound and carbohydrate metabolism”, “lipid fatty acid and isoprenoid metabolism” as well as “secondary metabolism” were predominantly enriched in both upregulated and downregulated gene sets (Fig 7). In contrast, categories related to mitochondrial function (respiration, electron transport, mitochondrion biogenesis and mitochondrial inner membrane) as well as protein synthesis (translation, ribosome biogenesis) were enriched only among Ros1-upregulated genes. About 170 genes belonging to “cell cycle and DNA processing”and 55 genes belonging to “cell growth and morphogenesis” were differentially regulated. Although they did not show significant enrichment, both categories were highlyrepresented in the upregulated gene set. Unexpectedly, Ros1 was also shown to cause a massive shift in secreted effector gene expression. 128 effectors genes were downregulated including 126 effector genes without functional domains, *cmu1* [8] and *UMAG_01130* [13], two effectors containing a functional domain. In addition, 70 effector genes were upregulated by Ros1: 68 without functional domains and two with functional domains, *UMAG_03615* [10] and *UMAG_11763* [13]) (Fig 8, S1 Table).

To identify the genes directly targeted by Ros1, we carried out a ChIP-seq analysis. Maize seedlings were infected with a compatible pair of complemented *ros1* deletion strains expressing an HA-tagged version of Ros1. A pair of compatible strains complemented with the native version of Ros1 without an HA tag was used as negative control. After sequencing the output DNA from three biological replicates, 1907 peaks showed high reproducibility, a significant peak shape score (> 20) and a low *p*-value (*p* < 0.01) (S2 Table). 1441 distinct intergenic regions including 620 intergenic regions for divergently transcribed genes were found to be targeted by Ros1. Only considering promoter regions, this brings the number of genes potentially targeted by Ros1 to at least 1913 (S3 Table).

**Fig 9:**
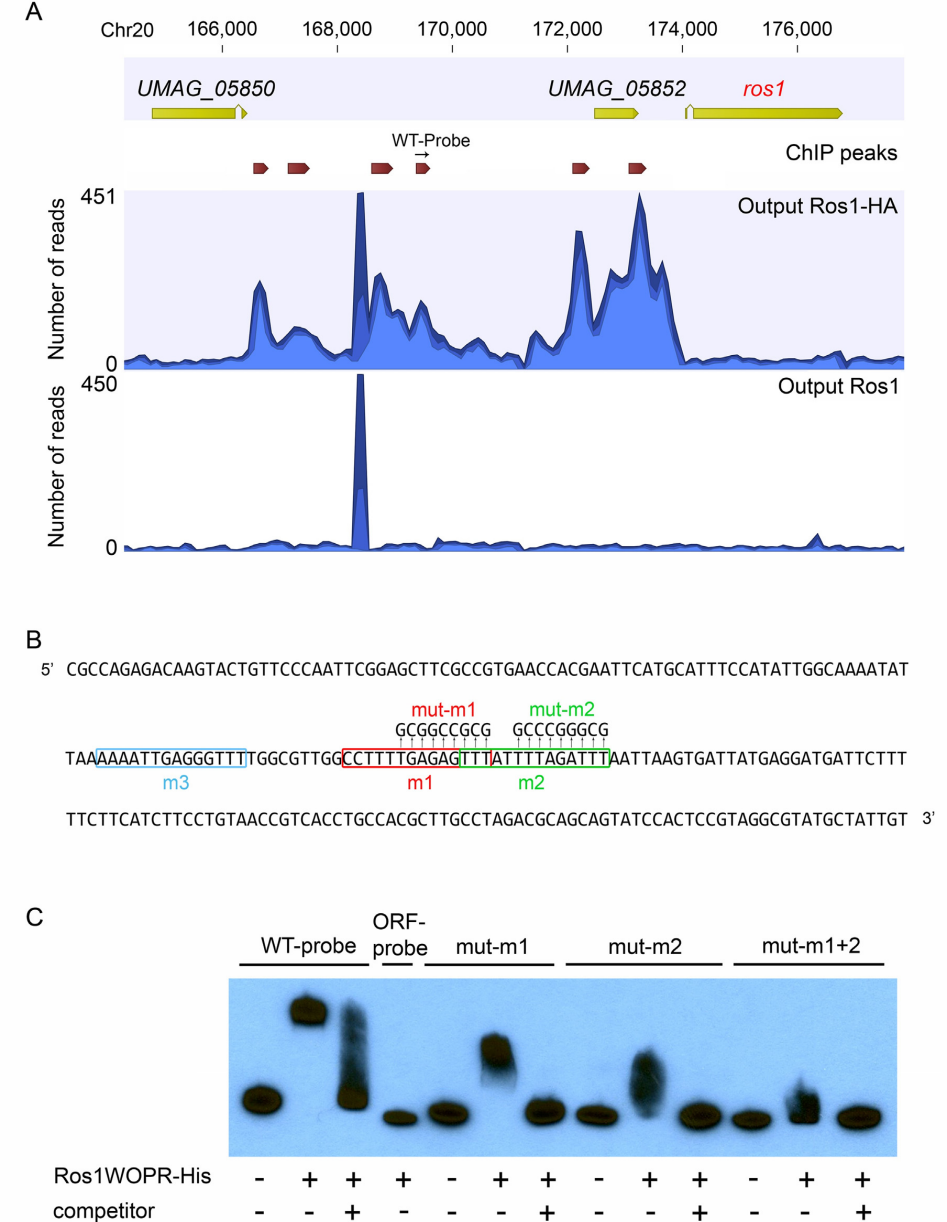
Ros1 binds to the *ros1* promoter region. (A) The graph generated with the CLC Genomics Workbench 7.5 software (CLC bio) shows the ChIP-seq read distribution in the genomic region containing *ros1* in output DNA from the sample where maize was infected with FB1Δros1-Ros1HA x FB2Δros1-Ros1HA (Output Ros1HA) and in the control sample where the infection was done with FB1Δros1-Ros1 x FB2Δros1-Ros1 (output Ros1) Open reading frames are represented by yellow arrows. Peaks with significant peak shape scores are indicated in the ChIP peaks lane by dark red arrows. The location of the fragment (WT-probe) used as a probe for EMSA is indicated by a black arrow. (B) Sequence of the probe fragment used for EMSA assays. Putative binding sites (m1, m2 and m3) for Ros1 are boxed and mutations introduced in the respective sites are indicated (mut-m1, mut-m2). (C) In vitro binding of Ros1WOPR-His to the *ros1* promoter. Ros1WOPR-His expressed and purified from *E*. *coli* was used in EMSA assays with the probe shown in B (WT-probe). When incubated with Ros1WOPR-His, the WT-probe was shifted and this could be competed by addition of a non-labeled WT-probe (competitor). Ros1WOPR-His did not bind a probe of the same length corresponding to a part of the *ros1* coding sequence (ORF-probe). Probes mut-m1 and mut-m2 harboring mutations in motifs 1 and 2 are also bound by Ros1WOPR-His, but the interaction results in a less pronounced shift than observed for the WT-probe and an even smaller shift when both motifs are mutated (mut-m1+2). Binding to mutated probes could also be efficiently competed by adding a non-labeled WT-probe.

Of the 2006 genes which were differentially expressed in the RNA-seq data, only 790 (40%) displayed at least one ChIP peak in their upstream region (S1 Table) suggesting that a significant part of Ros1 regulation depends on intermediate regulators. In total 80 transcription factor-encoding genes were differentially expressed in the RNA-seq dataset and of these 42 were downregulated and 38 were upregulated by Ros1. 25 upregulated transcription factor genes were predicted to be direct targets from the ChIP-seq analysis (S1 Table) and six of these including *ros1* displayed multiple Ros1-ChIP peaks in their promoters (S2 Table). For example, in the long intergenic region between *ros1* and *UMAG_05850* (Fig 9A) we detected six regions bound by Ros1, suggesting a rather complex regulation including autoregulation. The ChIP analysis also revealed that Ros1 binds the promoters of genes previously identified as regulators of sporogenesis *rum1*, *hgl1*, *tup1* and *ust1*, suggesting that they could be direct targets. These genes did not show differential regulation by Ros1 in the RNA-seq dataset generated at 8 dpi. However, a time-resolved analysis of their expression pattern showed that *rum1*, *hgl1* and *ust1* are slightly but significantly induced by Ros1 at 10 and 12 dpi (S4 Fig). In general, Ros1 binding is detected more frequently upstream of upregulated genes than upstream of downregulated genes (50% of the upregulated genes against 30% of the downregulated genes) (S1 Table). This suggests that Ros1 acts primarily as a transcriptional activator but can also function as a repressor. A more detailed discussion of the RNA-seq and ChIP-seq analysis is found in the discussion to avoid redundancy.

### In vitro DNA binding of Ros1

The WOPR regulators Wor1, Ryp1 and Mit1 all recognize a similar DNA binding motif [20, 24]. A search for the corresponding 14 bp consensus sequence in Ros1 ChIP-seq data (FIMO online tool, http://www.meme-suite.org), identified 975 motifs (p-value < 0,001) corresponding to 763 ChIP peaks (S4 Table). To test whether one of these regions is directly bound by Ros1, we carried out electrophoretic mobility shift assays (EMSA) using a recombinant His-tagged version of Ros1 containing the WOPR domain only (Ros1WOPR-His). As target we used a 237 bp biotinylated probe corresponding to the *ros1* promoter region between 2854 and 3090 bp upstream of the *ros1* gene (Fig 9A and 9B) containing three putative binding motifs (WT-probe). In presence of Ros1WOPR-His in a molar ratio of 2700:1 of protein to DNA the probe was completely shifted. This shift could be abolished by addition of a 500-fold excess of unlabeled WT-probe competitor, indicating that Ros1 interacts specifically with the WT-probe (Fig 9C). In comparison, incubation of Ros1WORP-His with a fragment of the same length from the *ros1* open reading frame (ORF-probe) did not lead to a mobility shift (Fig 9C). To narrow down the region bound by Ros1, mutations were introduced in the probe sequence. Two of the predicted Wor1-like binding motifs (m1 and m2) located at the center of the peak (Fig 9A), were mutated in probes mut-m1 and mut-m2 (Fig 9B) and tested for binding by Ros1WOPR-His. For both probes we observed discrete, significantly smaller shifts than for the WT-probe and the interactions could again be competed by an excess of unlabeled WT-probe (Fig 9C). When a fragment containing both mut-m1 and mut-m2 (mut-m1+2) mutations was used, an even less shifted complex was observed which could be competed (Fig 9C). This strongly indicates that the predicted m1 and m2 sites are indeed bound by Ros1 and suggests furthermore, that the WT-probe fragment contains an additional binding site, most likely the m3 site (Fig 9B). Using similar conditions, we also tested the binding of Ros1WOPR-His to other promoter regions identified by ChIP which contain at least one predicted binding site. Probes were designed for promoters of a gene encoding a transcription factor (*UMAG_02775*) upregulated by Ros1, four effector genes downregulated by Ros1 (*UMAG_02854*, *UMAG_04040*, *UMAG_02538* [10], *cmu1* [8]) and three effector genes upregulated by Ros1 (*UMAG_03138*, *UMAG_12258*, *UMAG_03046*). All probes were specifically bound by Ros1WOPR-His (S5 Fig), confirming that these genes are direct targets of Ros1.

### Phenotypic analysis of mutants lacking direct and indirect Ros1 target genes encoding transcription factors

**Fig 10:**
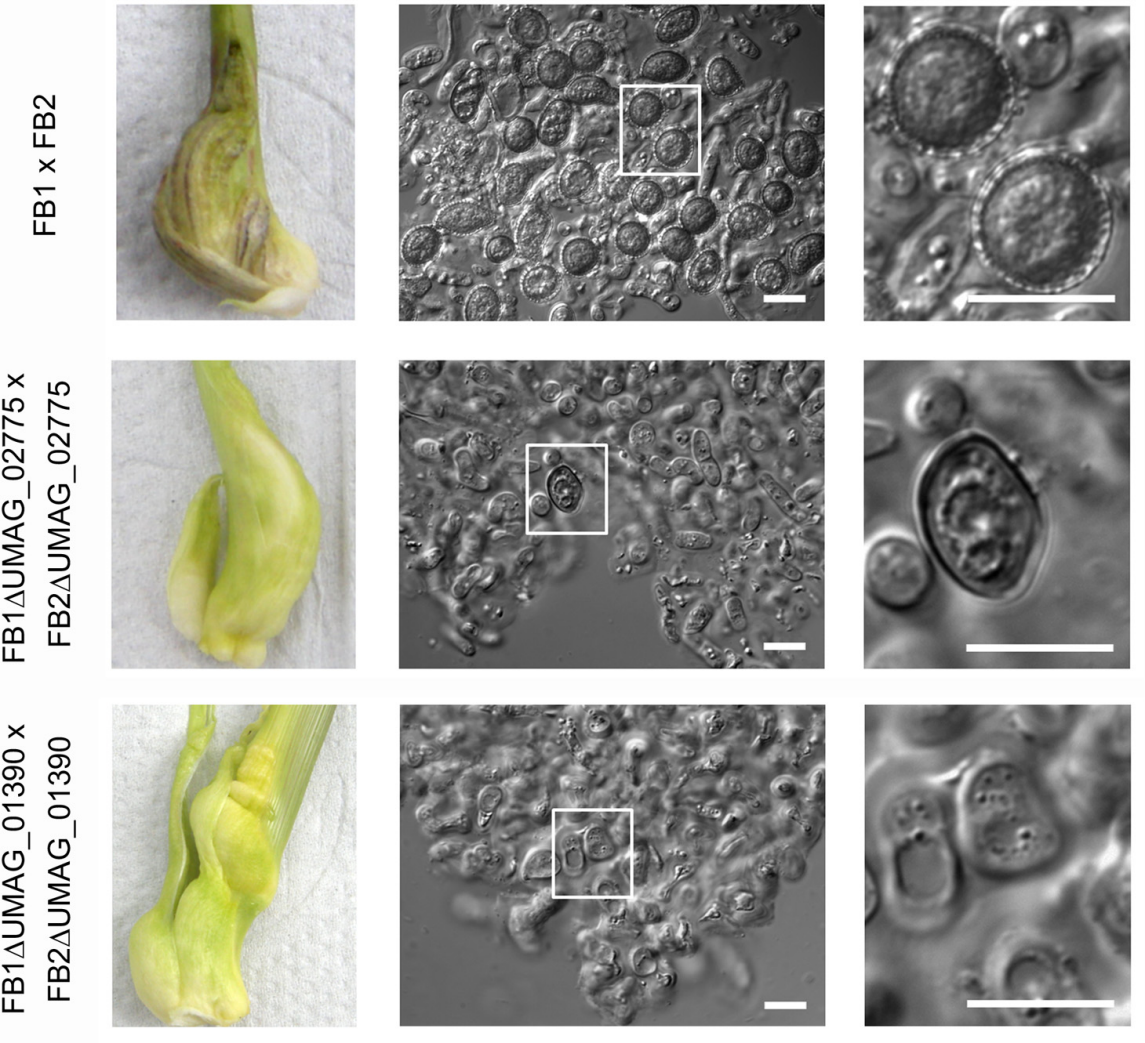
Two transcription factors targeted by Ros1 are involved in the regulation of spore development. Maize seedlings were inoculated with the indicated strains. Typical tumors induced by wild type and corresponding deletion strains are shown in the left panels. Tumor tissue was collected at 12 dpi, dispersed in water and spore structures were observed with a light microscope (middle panels). Enlarged parts of the middle panel are depicted in the right panel. Bar = 10 μm.

RNA-seq and Chip-seq analysis had shown that 47 transcription factor genes may represent direct targets of Ros1 while 33 may represent indirect targets. The expression pattern of two of these, *UMAG_02775* (presumed to be directly regulated by Ros1) and *UMAG_01390* (presumed to be indirectly regulated by Ros1) was additionally determined in maize plants infected with FB1 x FB2 or the corresponding *ros1* deletion strains in a time course experiment (S6 Fig). Results show that Ros1 is responsible for the late upregulation (between 8 dpi to 12 dpi) of these two genes.To follow up on these two transcription factors, the corresponding genes were deleted in FB1 and FB2 and mutant strains were then tested for virulence in maize seedlings and for their ability to produce teliospores (Figs 10 and S7). Tumor induction was not affected by the deletion of *UMAG_02775* and the mutant hyphae produced aggregates (Fig 10). However, in these aggregates only few spores developed and these were misshaped and were missing the ornamentation characteristic of mature spores (Fig 10).The deletion of *UMAG_01390* attenuated virulence to a comparable extent to what had been observed in the *ros1* mutant strains (Fig 10). Contrary to the *ros1* mutant, *UMAG_01390* deletion strains showed hyphal aggregation and reached the fragmentation stage of spore development (Fig 10). However, fragmented hyphal cells failed to enter the maturation process and did not give rise to ornamented teliospores (Fig 10). Taken together these results illustrate that *UMAG_02775* and *UMAG_01390* genes both affect discrete steps in spore development downstream of Ros1.

## Discussion

**Fig 11:**
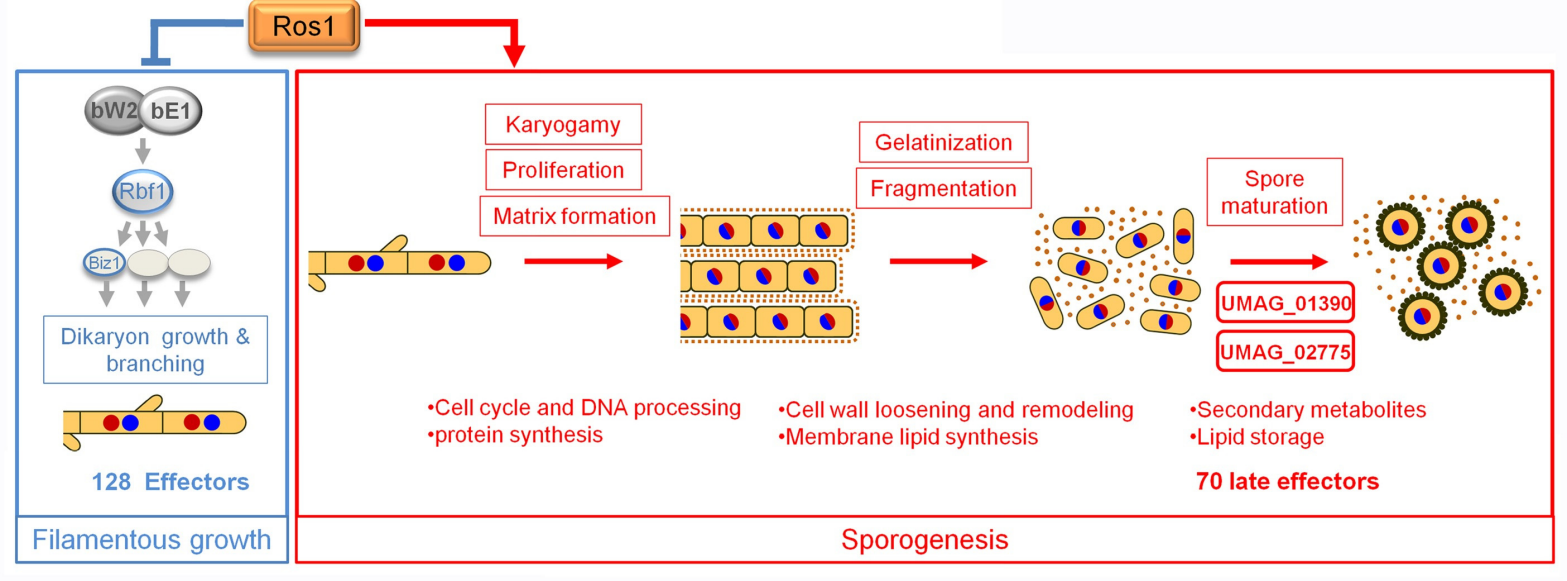
Model for Ros1 function in the control of late development of *U*. *maydis*. The model shows the processes repressed (blue box) and induced (red box) by Ros1 during the late development of *U*. *maydis*. Ros1 inhibits filamentous growth of the dikaryon by downregulating elements of the bE/bW regulatory cascade. It also represses 128 effector genes (126 of the 320 predicted effector genes [47, 48] without functional domain, *cmu1* [8] and *UMAG_01130* [13]) (blue box). Concomitantly, Ros1 induces teliospore formation (Red box). Ros1 triggers karyogamy followed by proliferation of the diploid and matrix formation. This coincides with the upregulation of genes involved in cell cycle, DNA processing and protein synthesis. The gelatinization of the cell wall and subsequent fragmentation of sporogenous hyphae might involve cell wall loosening and membrane lipid synthesis. The two Ros1 induced transcription factors UMAG_02775 and UMAG_01390 regulate spore maturation following the fragmentation stage. Secondary metabolism, lipid storage as well as 70 late effectors are induced by Ros1. Yellow = cytoplasm, black line = cell wall, blue / red discs = nuclei, brown dots = matrix.

In this study we demonstrate that late biotrophic development in *U*. *maydis* is coordinated by Ros1, a member of the WOPR family of fungal regulators. Ros1 does not influence the ability of *U*. *maydis* to induce tumor formation, but is the key regulator for switching from *b*-dependent filamentation to hyphal aggregation and spore formation. This development is accompanied by a dramatic shift in the expression of 60% of the putative effector genes without functional domain [47, 48]. The processes regulated by Ros1 are depicted schematically in Fig 11.

### Ros1 and the regulation of late biotrophic development

Previous light microscopy studies had seen paired nuclei commonly in hyphae outside the aggregates but not in the aggregates of sporogenous hyphae [5]. This is consistent with our analysis using fluorescent nuclear markers which shows that hyphae in aggregates are monokaryotic. In contrast to wild type strains, *ros1* deletion strains are unable to form such aggregates, fail to accumulate matrix material and remain dikaryotic. This could suggest that karyogamy precedes the formation of hyphal aggregates and may be required to initiate the synthesis of the mucilaginous matrix (Fig 11). In wild type strains hyphal aggregates develop within the plant intercellular space and considerably expand over time due to a dramatic increase of fungal biomass which is not observed in plants infected with *ros1* deletion strains. When expressed ectopically in axenic culture, Ros1 triggered mitotic divisions suggesting that aggregate expansion during colonization is due to multiple rounds of mitotic divisions of the diploid cells as was earlier hypothesized [5]. Moreover, the phenotype of cells expressing *ros1* prematurely in hyphae indicates that the resulting cell divisions do not involve clamps. We speculate that without clamps diploid cells proliferate faster than the dikaryon which could explain the rapid and massive increase of fungal biomass late in infection.

### Ros1 triggers cell wall and membrane rearrangements

Starting at six dpi *U*. *maydis* cells begin to aggregate and form large, ball-like structures. What glues cells together is presently unknown, but based on the finding that the matrix can be stained with basic fuchsin [49], it is likely that the matrix contains polysaccharides. In addition, it was reported that hyphae containing diploid nuclei are partially refractory to chemical fixation and this was attributed to lysis of the cell wall and its conversion to a gelatinous material [5]. Incidentally we noticed that during sporogenesis staining of the cell wall, but not of the septa, with wheat germ agglutinin-Alexa Fluor 488 becomes fainter with time (Fig 2, 8 dpi time point) which could reflect chitin degradation or modification of the cell wall. Cell wall loosening might facilitate the changes in cell morphology which are associated with hyphal fragmentation and spore maturation. The matrix could connect the cells and shield them against biotic and abiotic stresses. Among the 255 Ros1-regulated genes belonging to the functional category “C compound and carbohydrate metabolism”, 55 are predicted to be involved in polysaccharide metabolism. Most of them encode glycoside hydrolases. They are enriched in both up and downregulated gene sets. The two most upregulated genes encode enzymes targeting the fungal cell wall, an EXG1 beta-glucanase (FC = 831) and a chitinase A (FC = 441) (S1 Table), and both could conceivably be involved in the gelatinization process. The strong Ros1-dependent upregulation of an UDP glucose dehydrogenase (*UMAG_00118*) suggests an increased glucuronic acid production at the onset of sporogenesis. Bacterial extracellular matrices as well as the capsule of *Cryptococcus neoformans* contain highly polar glycosaminoglycans which are rich in glucuronic acid [50]. Among the genes upregulated by Ros1, we found a gene related to *C*. *neoformans* CAP59. This *C*. *neoformans* gene is involved in capsule synthesis by supporting polysaccharide export [51]. It is conceivable that the related gene in *U*. *maydis* (*UMAG_11017*) could fulfill a similar function in the export of matrix material. We also observed that the repellent gene *rep1*, which is already upregulated in hyphae [52] is further induced by Ros1 during sporogenesis. Repellents are structural proteins forming amyloid fibrils at the cell surface which mediate hyphal adhesion to hydrophobic surfaces [53]. These amyloid fibrils could be of importance during sporogenesis, could aid in connecting hyphae with each other and assume a structural function in the formation of the matrix. Consistent with this hypothesis hydrophobins which are functionally similar to repellents [54] play a structural role in the formation of fruiting bodies [53]. Since *rep1* deletion mutants still produce viable teliospores [52] Rep1 is unlikely to be essential for teliospore formation in *U*. *maydis*. However, it cannot be excluded that the efficiency of spore formation is affected in *rep1* mutants.

In addition to genes involved in cell wall modification we observed the Ros1-dependent upregulation of several genes involved in the synthesis / modification of membrane lipids (Fig 10). Among them were genes encoding ergosterol biosynthetic enzymes, sphingolipid biosynthetic enzymes and fatty acid synthesizing / modifying enzymes like cyclopropane fatty acid synthase (S1 Table). The likely ensuing alterations of plasma membrane composition might reflect that the plasma membrane in spores has a different composition from that of vegetative cells. In *Schizosaccharomyces pombe* and *S*. *cerevisiae*, sporulation involves de novo synthesis of the forespore membrane within the cytoplasm of mother cells, which subsequently becomes the plasma membrane of the developing ascospores [55]. Cyclopropane fatty acid synthesis was also reported to be essential for fruiting body development in the basidiomycete *Coprinus cinerea* [56]. Sphingolipids have important roles in membrane and lipoprotein structure and in cell regulation as signaling molecules for growth and differentiation. They have been shown to be required for proper cell growth and morphology in *U*. *maydis* [57] and the upregulation of sphingolipid synthesis by Ros1 might be prerequisite for teliospore differentiation. Many genes involved in fatty acid beta oxidation were downregulated by Ros1. This may reflect that the predominant spore storage fatty acids of *U*. *maydis* are linoleic and palmitic acid [58]. In line with this, a gene encoding a caleosin-like protein (*UMAG_02753*) is strongly upregulated by Ros1 (FC = 220). Caleosins are involved in the structural maintenance and turnover of lipid storage organelles, so-called lipid droplets [59]. The strong upregulation of this gene at 8 dpi might thus indicate lipid storage in spores.

### Ros1 and its relation to other members of the WOPR family

Most WOPR regulators characterized so far in plant pathogenic ascomycetes regulate both plant invasion and sexual / asexual spore production [26–28, 30–32]. Ros1 is the first WOPR protein characterized in a basidiomycete. In comparison to Wor1 from *C*. *albicans* and Ryp1 from *Histoplasma capsulatum* for which ChIP-chip identified only about 200 and 700 targets [60, 61], Ros1 might directly regulate a much larger set of genes (1900 identified by ChIPseq). Binding sites for all WOPR proteins characterized to date are conserved [20, 24], and we have shown here that Ros1 can also bind the 14 bp consensus sequence identified for Wor1 [20]. However, not all Ros1-bound regions identified by ChIP-seq (765 out of 1913) harbor this motif. This could indicate that Ros1 can recognize additional sequences more distantly related to the Wor1 binding site or that it can bind additional sequences via interaction with other transcription factors. Recent studies in *C*. *albicans* and *H*. *capsulatum* have provided evidence that WOPR proteins can bind promoters in complex with other core regulators and have suggested that the formation of these complexes might be mediated by glutamine-rich regions [61, 62] which serve as protein interaction domains also in other proteins [63]. The presence of an exceptionally long poly-glutamine tract in the C-terminal domain of Ros1 might reflect such a role. Similarly to Wor1 [60] and based on finding that direct Ros1 targets include both up and downregulated genes Ros1 may function both as an activator and a repressor.

In *C*. *albicans*, *S*. *cerevisiae* and *H*. *capsulatum*, Wor1, Mit1 and Ryp1 are part of core regulatory networks in which each transcription factor regulates and is regulated by the others [24, 61, 62]. In these networks, WOPR regulators fulfill a critical function because they bind most of the target promoters [23, 24, 61, 64]. Similar to the genes encoding core regulators in these species, *ros1* exhibits an unusually long promoter and binds to its own promoter, most likely reflecting autoregulation. Moreover, Ros1 directly regulates many transcriptional regulators which remain to be investigated and which could potentially be core regulators.

WOPR regulated genes have functionally diverged considerably during evolution and show very poor overlap even in closely related species [24]. However, several classes of genes / processes regulated by WOPR proteins in plant pathogenic fungi appear conserved: these include spore formation as well as secondary metabolism and effector gene expression (all discussed below).

### Ros1 downregulates components of the bE/bW cascade

Premature expression experiments in hyphae showed that Ros1 is antagonistic to bE/bW and inhibits *b*-dependent filamentation (Fig 11). Analysis of Ros1 dependent gene expression revealed that the negative effect of Ros1 on filamentous growth could originate from an alteration of the bE/bW regulatory cascade. About 50% of the 345 genes which are regulated by overexpressing bE1/bW2 in axenic culture [3] were found to be affected by Ros1. 75 genes upregulated by bE/bW are repressed by Ros1 and this includes several regulators of the *b* cascade: Rbf1, the central regulator of pathogenic development responsible for inducing the majority of the 345 *b*-regulated genes, and two of its downstream targets, Biz1 and Hdp1, which modulate the cell cycle and regulate the growth of filaments [3, 65]. Conversely, 40 genes downregulated by bE/bW are induced by Ros1 and 36 genes upregulated by bE/bW were also induced by Ros1. Among the 170 *b*-dependent genes differentially expressed, Ros1 is likely to directly regulate 71. Interestingly, *bE*, *bW* and *prf1*, the main regulators of the *b* cascade, are only slightly repressed by Ros1, suggesting that the Ros1 induced inhibition of filamentation does not re-establish the budding program This is also apparent when *b*-expressing cells are microscopically observed after premature expression of Ros1. In these cases we observe the formation of septated, compartmentalized cells each containing a single nucleus. Such structures, where cell segments containing a single nucleus become deliminated by thick septa, are reminiscent to structures in sporogenous hyphae [5]. This suggests that Ros1 targets the *b* cascade at certain nodes without downregulating the entire cascade and this may then be prerequisite for entering this septation program. During infection, inhibition of parts of the *b* cascade would occur at a specific stage of biotrophic development, when the cell cycle has been released and the *U*. *maydis* dikaryon is proliferating in the infected tissue by clamp formation. This developmental stage is different from the filamentous stage achieved by overexpressing *bE1/bW2* in axenic culture. Therefore, data sets generated by Heimel *et al*. (2010) [3] and the 8 dpi time point studied here after infection with the dikaryon are not fully comparable.

### Ros1 regulates karyogamy and subsequent cell proliferation

Our studies have illustrated that *ros1* mutants do not initiate nuclear fusion and fail to trigger massive proliferation. This shows for the first time that the strong increase in fungal biomass late in infection may require karyogamy to be completed. An inspection of the RNA-seq data revealed no differential regulation by Ros1 of the four *U*. *maydis* genes related to genes *KAR7*, *KAR2*, *KAR3* and *KAR4* implicated in karyogamy in *S*. *cerevisiae*. While 168 genes involved in DNA processing and cell cycle (Fig 11) were differentially regulated by Ros1, we did not observe any significant enrichment for these categories in the *ros1* upregulated gene set. One likely explanation is that at the 8 dpi timepoint chosen for the RNA-seq analysis genes involved in karyogamy are already shut off. Among the 89 Ros1 upregulated genes in the category “DNA processing and cell cycle” we detect clear indicators for proliferation like DNA helicases, DNA primase, PCNA and several DNA mismatch repair proteins (S1 Table). However, the *ros1* deletion strain is also able to replicate its DNA in the dikaryotic phase, and this could explain why no significant enrichment is observed for the category “cell cycle and processing” (Fig 7). In line with this interpretation, protein synthesis and mitochondrial function (respiratory chain) were both overrepresented functional categories among the genes upregulated in sporogenous hyphae compared to the dikaryon of the *ros1* mutant. Late proliferation of diploid hyphae might rely primarily on plant sugars as suggested by the strong upregulation of several glycoside hydrolases located at the surface of the cells, e.g. a secreted trehalase (*UMAG_02212*), a membrane located glucoamylase (*UMAG_04064*), the secreted invertase SUC2 and several other non characterized secreted glycoside hydrolases (e.g. *UMAG_00102*, *UMAG_06434*).

Infection experiments in maize seedlings showed that *ros1* mutants cause significantly fewer dead plants compared to the wild type. We speculate that the massive late proliferation of the wild type in hyphal aggregates might negatively affect plant fitness and account for the high percentage of plants not surviving under our glasshouse conditions.

### Ros1 and the regulation of spore development

We have shown that Ros1 controls the early events of spore development by inhibiting *b*-dependent filamentation and inducing karyogamy and hyphal aggregate formation as well as the initiation of spore formation. Among the upregulated genes in wild type infections are 38 putative transcription factors which are Ros1-regulated. Mutants in two of these transcription factors genes (*UMAG_02775* and *UMAG_01390*) revealed that both mutant strains were able to reach the gelatinization stage and to trigger hyphal fragmentation but failed at discrete subsequent steps of spore maturation. This suggests that these genes are regulators of the spore maturation process downstream of Ros1. Other regulatory proteins previously shown to interfere with teliospore formation are Hgl1, the histone deacetylase Hda1, and the transcription factors Rum1, Ust1, and Tup1 [33, 43, 66–68]. Hgl1, Rum1 and Tup1 are all required for proper spore development after the fragmentation stage [33]. Ust1 (*UMAG_15042*) is a transcriptional repressor in haploid cells which represses filamentation as well as formation of spore-like structures. [68, 43]. The ChIP analysis revealed that Ros1 binds the promoters of *rum1*, *hgl1*, *tup1* and *ust1*, suggesting that they could be direct targets. *rum1*, *hgl1* and *ust1* were slightly but significantly induced by Ros1 at 10 and 12 dpi (S4 Fig). The small effect of Ros1 could indicate that these genes are regulated by a combination with other TFs. For *rum1* and *hgl1* this is in line with their requirement for spore formation. To explain the upregulation of the negative regulator *ust1* by Ros1, we consider its role in controlling the budding program [68] may be required during sporogenesis when we observe its upregulation. This would imply that the observed formation of spore-like structures in haploid cells of the *ust1* mutant [68], could be a default pathway and not reflect what is happening during the sporulation program after infection. Consistently, we do not observe expression levels of *ust1* during the *U*. *maydis* life cycle which are below the levels in axenic culture.

*tup1* expression levels were not influenced by Ros1. As we did not find evidence that Ros1 differentially regulates the divergently transcribed *UMAG_10827* gene (S1 Table), we speculate that Ros1 binding to the promoter is not essential for the activity of this promoter under the tested conditions. Having shown that Ros1 participates in regulating *hgl1* and *rum1* expression and having shown that Ros1 is required at an earlier stage that precedes the formation of the sporogenous hyphal aggregates prior to karyogamy places Ros1 upstream of these genes and processes and highlights the importance of Ros1 for the regulation of sporogenesis. The functional analysis of the other Ros1-regulated transcription factors identified here is a promising avenue to elucidate the entire regulatory network controlled by Ros1 during sporogenesis.

### Ros1 and the control of secondary metabolism

Secondary metabolism is commonly associated with sporulation processes in microorganisms, including fungi [69]. Among the genes upregulated by Ros1, we found *pks1* and laccase I responsible for the synthesis of the melanin pigment in teliospores [43]. In addition, the most upregulated gene involved in secondary metabolism is related to versicolorin B synthase, an enzyme involved in the synthesis of aflatoxine in *Aspergillus* sp. [70]. In *U*. *maydis* this gene belongs to a newly identified gene cluster (E. Reyes-Fernàndez and M. Bölker, personal communication) and we speculate that it is responsible for an intermediate step in the biosynthesis of an antimicrobial compound and / or a pigment.

A gene cluster involved in the synthesis of itaconic acid [71] is also upregulated by Ros1 and in this case it appears to be the regulatory gene *ria1* which is directly targeted while the promoters of the other genes in the cluster are not bound by Ros1. Itaconic acid inhibits the bacterial glyoxylate shunt essential for many bacteria to survive during infection of mammalian hosts. The gene cluster for the production of the mannosylerythritol lipids which have biosurfactant and antimicrobial activity [72] is also upregulated by Ros1. The antimicrobial properties of itaconate and mannosylerythrol lipids might serve to combat against competing microbes during late stages of *U*. *maydis* development.

### Ros1 is a major regulator of effector genes late in infection

The modulation of effector gene expression seems to be a common trend shared by many WOPR proteins from various plant pathogenic fungi [26, 29–32]. However, in *C*. *fulvum* and *V*. *dahliae* the deletion of the respective WOPR protein is associated already with growth defects in axenic culture [31, 32], and in *Zymoseptoria tritici* abnormally swollen cell structures are observed during axenic growth in the *wor1* mutant [30]. This suggests that in these cases the respective WOPR protein might be more involved in developmental processes than in specific regulation of effector genes. Furthermore, in cases where effector gene expression has been analyzed, effectors are usually upregulated by the respective WOPR protein and the upregulation concerns a relatively small number of effectors (six in *F*. *oxysporum*, 14 in *F*. *verticilloides*, six in *V*. *dalhiae*) [26, 29, 32]. One of the main findings emerging from our transcriptional analysis is that Ros1 in *U*. *maydis* induces a massive switch in the effector repertoire affecting about 60% (194 genes) of the predicted set of effector genes without functional domains [47, 48] as well as 4 genes encoding effectors with functional domain. 82 of the 198 differentially regulated effector genes are likely to be directly targeted by Ros1. These include *cmu1* [8], *pit2* [15, 73], *mig1* [74], 23 genes residing in effector clusters (2A, 2B, 5A, 6A, 10A, 19A, 22A,) [10] as well as 45 of the late-induced effectors. The differential regulation of the genes which are not direct targets likely results from the alteration of the bE/bW cascade by Ros1. Contrary to Sge1 which induces effector genes required for virulence in *F*. *oxysporum* [26], Ros1 mostly downregulates effector genes. 26 of the 128 downregulated effector genes (Fig 7) reside in clusters where the cluster deletion causes a virulence defect (5A, 5B, 6A, 10A, 19A) [10] and 10 are members of the *eff1* effector family that also contributes to virulence [75]. Unexpectedly, among the effectors downregulated by Ros1 are also critical effectors involved in the inhibition of plant defense responses like Stp1, Cmu1 and Pit2 [8, 15, 73, 76]. In addition, of the 14 leaf-specific effector genes without functional domain [13] (of which many contribute to virulence) all are downregulated by Ros1. While it is easy to conceive that *see1*, an effector involved in cell expansion associated with tumor formation [16] is downregulated because tumor formation has happened already at the stage when *ros1* is induced, the downregulation of effectors with critical functions in plant defense suppression is more difficult to explain. Either plant defenses at this late stage of *U*. *maydis* development could be distinct from the early stages of infection and require a different effector set for suppression. Or alternatively the massive production of matrix material which is associated with the formation of the fungal aggregates, could shield the aggregated hyphae from plant detection or from the action of antimicrobial compounds associated with plant defenses. Reduced effector expression could then be sufficient to maintain the inhibition of plant defense at the periphery of the aggregates.

As a third possibility it could be the combined action of late induced effectors plus matrix that is effective. In total, there are 70 effector genes that are induced by Ros1 (68 without functional domains). Four of them (*UMAG_03138*, *UMAG_05926*, *UMAG_03046*, *UMAG_12258*) exhibited a 50-fold higher expression in infections with wild type compared to the *ros1* mutant strains. 13 of these late-induced effector genes reside in effector clusters (19A, 10A, 2B, 5A, 6A, 9A) including clusters involved in virulence (5A, 6A, 10A, 19A) [10]. Since the cluster deletions were all generated in a solopathogenic haploid strain [10] in which spore formation does not follow the same sequence of events as in the dikaryon, we cannot presently assess whether these late effectors affect processes facilitating *U*. *maydis* sporulation, inhibit late plant defense responses and / or are participating to the formation of the matrix. Alternatively, these late effectors might, together with secondary metabolites induced at that stage, be used as a cocktail to defend the spores against other microbes which could colonize tumor tissue when it dries up and ruptures, releasing the spores. One could also consider that late effectors could fulfill a signaling function inside the aggregates to control the spore maturation process.

In conclusion, Ros1 emerges as the central regulator of a major developmental reprogramming leading to teliospore production and completion of the life cycle in *U*. *maydis* (Fig 10). Of particular interest is how *U*. *maydis* can survive in the hostile plant environment with reduced expression of a large set of effector proteins of which many have a critical virulence function early during colonization. In addition, elucidating the role of the “late effectors” which are specifically induced by Ros1 promises to provide new insights into how this facultative biotrophic fungus has established itself in its natural environment. Moreover, we are confident that deciphering the structure and dynamics of the regulatory network in which Ros1 functions will provide an understanding how this master regulator achieves such a broad control over gene expression. Another yet unresolved task is the identification of the upstream signals triggering *ros1* induction during biotrophic development and thereby inducing late development including spore formation.

## Materials and Methods

### Strains and growth conditions

The *Escherichia coli* strain Top10 (Life technologies) and BL21(DE3)pLysS (Promega) were used for cloning purposes and for expression of recombinant Ros1 protein respectively. *U*. *maydis* strains used in this study are listed in S5 Table, they are derivates of haploid strains FB1 and FB2 [77] or AB33 [39]. Cells were grown in liquid YEPSL (0.4% yeast extract, 0.4% peptone, 2% sucrose) at 28°C on a rotary shaker at 220 rpm. For virulence assays, compatible haploid strains were grown separately in YEPSL to an OD_600_ of 1.0, transferred to the same volume of sterile water and mixed in equal amounts prior to injection into maize seedlings.

For premature *ros1* expression studies, AB33 and strains derived from AB33 were grown in complete medium (CM) [78] supplemented with glucose (2%) to an OD_600_ of 0.5. Cells were collected by centrifugation, washed with H_2_O and resuspended in nitrate minimal medium (NM) [78] containing arabinose (2%) as sole carbon source. Cells were subsequently grown for 12h for microscopic observation. To induce *ros1* after the switch to filamentous growth, AB33 or AB33 derived strains were incubated for 6 h in NM + glucose and then shifted to NM + arabinose for 6 h. All chemicals used for media preparation were of analytical grade and were obtained from Sigma-Aldrich.

### Construction of *U*. *maydis* strains

PCR reactions were performed using the Phusion High-Fidelity DNA Polymerase (New England Biolabs). Templates were either FB1 genomic DNA or indicated plasmid DNAs. Point mutations were generated using the Quick change lightning kit (Agilent Technologies). Restriction enzymes were all supplied by New England Biolabs. *U*. *maydis* was transformed by protoplast-mediated transformation [79] Gene replacements and integrations into the *ip* locus [80] were verified by Southern blot analysis.

All primer sequences used to generate plasmids are listed in S6 Table. To generate the Ros1mCherry fusion construct, plasmid p123 [81] conferring resistance to carboxin and allowing integration into the *U*. *maydis ip* locus was used. mCherry-HA was amplified from plasmid p1139 (kindly provided by A. Djamei) using primers mCherry_EcoF / mCherry_NotR and cloned in place of *gfp* between EcoRI and NotI sites of p123 to generate pPotef-mCherry-HA. The *ros1* open reading frame was amplified with primers ros1_XmaF / ros1_EcoR and cloned between sites XmaI and EcoRI of pPotef-mCherry-HA to generate pPotef-ros1-mCherry-HA. pPotef-ros1-mCherry-HA was linearized by SspI prior to transformation *of U*. *maydis*. For the deletion of *ros1*, a PCR-based strategy [82] and the SfiI insertion cassette system [79] were used. 1 kb long left border and right border fragments adjacent to *ros1* were PCR-amplified using primer pairs Dros1LB_F/R and Dros1RB_F/R and FB1 genomic DNA as template. The resulting fragments were ligated to the hygromycin resistance cassette of pBS-hhn [82] via SfiI restriction sites and cloned into pCRII-TOPO (Life technologies) to generate pDros1. The deletion construct was PCR amplified from plasmid pDros1 using primers Dros1_F/R and transformed into *U*. *maydis* strains FB1 and FB2 to generate FB1Δros1 and FB2Δros1. The drag and drop cloning method [83] was used to generate plasmids pDUMAG_02775 and pDUMAG_01390 for the deletion of *UMAG_02775* and *UMAG_01390*, respectively. Primer pairs D02775LB_F/R and D02775RB_F/R and primer pairs D01390LB_F/R and D01390RB_F/R were used to amplify left and right border fragments from *UMAG_02775* and *UMAG_01390*, respectively. Left and right border fragments and the hygromycin resistance cassette were integrated in plasmid pSR426 (kindly provided by S. Reissmann) by homologous recombination in *S*. *cerevisiae*. The deletion constructs were excised from plasmids pDUMAG_02775 and pDUMAG_01390 after cleavage by Bsu36I and used for transformation of FB1 and FB2 to generate strains FB1ΔUMAG_02775, FB2ΔUMAG_02775, FB1ΔUMAG_01390 and FB2ΔUMAG_01390.

For complementation of *ros1* mutant strains, plasmid p123-Bsu was generated from plasmid p123 [81] by introducing a silent point mutation in the *cbx* gene to create a Bsu36I site using primers p123Bsu_F/R. The 7.5 kb long genomic region separating *ros1* from the upstream gene *UMAG_05850* was used as promoter in complementation constructs. This region might contain an additional gene (*UMAG_05852*). To make sure that complementation constructs would not have two copies of this gene, the putative start codon of *UMAG_05852* was deleted. To this end, the 6 kb intergenic region between *UMAG_05850* and *UMAG_05852* was amplified from genomic FB1 DNA with primers Cros1_KpnF / Cros1_SbfR and a second fragment containing *UMAG_05852* and *ros1* open reading frame was amplified using primers Cros1_SbfF / Cros1_NotR. Both fragments were ligated and cloned into pCRII-TOPO (Life technologies). In the resulting plasmid pCRII-TOPOros1 the sequence GCTGACGCATG containing the start codon of *UMAG_05852* is changed to TAGCATAG. Using primers TOPOros1_mF / TOPOros1_mR, a silent point mutation was introduced in pCRII-TOPOros1 to remove a KpnI site located in *UMAG_05852*. The complementation construct was then excised from pCRII-TOPOros1 and cloned into p123-Bsu between KpnI and NotI sites. The resulting plasmid pCros1 was linearized with Bsu36I and transformed into *ros1* deletion strains to generate strains FB1Δros1-ros1 and FB2Δros1-ros1.

For complementation of *UMAG_02775* mutant strains, fragments corresponding to the *UMAG_02775* promoter and open reading frame were PCR amplified from FB1 genomic DNA using primer pairs P02775_F/R and orf02775_F/R respectively and cloned in p123 between KpnI and NotI Sites. The resulting plasmid pCUMAG_02775 was linearized by SspI and transformed into FB1ΔUMAG_02775 and FB2ΔUMAG_02775 to generate strains FB1ΔUMAG_02775-UMAG_02775 and FB1ΔUMAG_02775-UMAG_02775.

For complementation of *UMAG_01390* mutant strains, a fragment corresponding to *UMAG_01390* promoter followed by the open reading frame was PCR amplified from FB1 genomic DNA using primer pairs C01390_F/R and cloned in p123 between KpnI and NotI sites. The resulting plasmid pCUMAG_01390 was linearized by SspI and transformed into FB1ΔUMAG_01390 and FB2ΔUMAG_01390 strains to generate strains FB1ΔUMAG_01390-UMAG_01390 and FB1ΔUMAG_01390-UMAG_01390.

For the generation of strains expressing a C-terminal Ros1HA fusion for the ChIP analysis, the *ros1* open reading frame without the stop codon was amplified from FB1 genomic DNA using primers Ros1_XmaF / Ros1_NotR and subcloned in vector p1306 (Kindly provided by A. Djamei) between XmaI and NotI sites upstream of a triple HA sequence to generate pRos1-3HA. A fragment containing the last 1 kb of *ros1* orf and the triple HA tag sequence was then amplified from pRos1-3HA using primers Endros1_NcoF / Endros1_PspR and cloned in pCros1 between NcoI and NotI sites. The resulting vector pCros1-3HA was linearized by Bsu36I and transformed in *ros1* deletion strains to generate FB1Δros1-Ros1HA and FB2Δros1-Ros1HA.

To allow expression of *ros1* from the *crg1* promoter, P_*crg1*_ was integrated upstream of *ros1* in the native locus of AB33 using pRU11 [39]. Right border (corresponding to the first 1000 bp of *ros1*) and left border fragments (corresponding to the 1000 bp upstream of the *ros1* start codon) were amplified from FB1 genomic DNA using primer pairs crg-ros1LB_F/R and crg-ros1RB_F/R, respectively. The generated fragments were cloned between NdeI and EcoRI sites of plasmid pRU11. The resulting plasmid pPcrg1-ros1 was linearized with NcoI and transformed into AB33 to generate strain AB33_Pcrg1_Ros1.

To express *ros1* from the *mig2-6* promoter, P_mig2-6_ [42] was amplified from FB1 genomic DNA using primers Pmig2-6_NdeF / Pmig2-6_XmaR and cloned into NdeI and XmaI sites of p123 to generate pPmig2-6. The *ros1* gene was amplified with primers Ros1_XmaF / Ros1_NotR and cloned via XmaI and NotI sites downstream of P_mig2-6_ in pPmig2-6. The resulting plasmid pPmig2-6-ros1 was linearized by SspI and transformed into *ros1* deletion strains to generate strains FB1Δros1_Pmig2-6_Ros1 and FB2Δros1_Pmig2-6_Ros1.

To allow expression of the Sso1 nuclear membrane marker fused to mCherry, P_sso1_ was amplified from FB1 DNA with primers Psso1_KpnF and Psso1_NcoR. P_otef_ was replaced by P_sso1_ in p123 using KpnI and NcoI sites to generate pPsso1. A fragment containing the mCherrySso1 fusion gene followed by the terminator of *sso1* (T_sso1_) was amplified from plasmid pBS-otef-mCherry-sso1-hyg [84] using primers McSso1_NcoF and McSso1_HpaR. The fragment containing mCherrySso1-T_sso1_ was then integrated between NcoI and HpaI sites in place of GFP-T_*nos*_ of pPsso1 to generate pPsso1-mcherry-sso1. This vector was linearized by SspI and transformed into FB1, FB2, FB1Δros1 and FB2Δros1. To introduce the nuclear marker nucleoporin Nup107 fused to GFP in the resulting strains, pNup107GFP-ble [85] was used. The insert from this plasmid encoding the Nup107GFP including the regulatory sequences was amplified with primers Pnup_F/R and integrated in the native nup107 locus to generate strains FB1_Pnup107_Nup107eGFP-_Psso1_Sso1mCherry, FB2_Pnup107_Nup107eGFP-_Psso1_Sso1mCherry and the corresponding *ros1* deletion strains. The sequence of all PCR-amplified regions was verified.

### Virulence assays

Haploid strains were grown in YEPSL medium to an OD_600_ of 1.0, washed and resuspended in sterile H_2_O. Compatible strains were mixed in a 1:1 ratio and syringe-inoculated into seven-day-old maize seedlings of the variety Gaspé Flint (originally provided by B. Burr, Brookhaven National Laboratories). Three independent infections were carried out for each strain and disease symptoms were evaluated after 12 days according to established disease rating criteria [10]http://journals.plos.org/plospathogens/article?id=10.1371/journal.ppat.1004272—ppat.1004272-Kamper1. Significant virulence differences between strains were assessed by one-way ANOVA applying the Tukey-Kramer test [36].

### RNA isolation

Total RNA was extracted from cells grown in axenic culture or from infected plant samples. Leaf material was ground in liquid nitrogen to a fine powder while cells in culture were pelleted by centrifugation and frozen in liquid nitrogen. Samples were resuspended in TRIzol reagent (Life technologies) and homogenized in a FastPrep-24 (MP Biomedicals). Total RNA was isolated according to the manufacturer's protocol. Genomic DNA contaminants were eliminated using the Ambion Turbo DNA free Kit (Life Technologies). For RNA sequencing RNA samples were further purified using the RNeasy Mini Kit (Qiagen) and the RNA quality was controlled using an Agilent 2100 Bioanalyzer.

### RNA sequencing

Total RNA samples from plants infected in three biological replicates with FB1 X FB2 strains or the corresponding *ros1* deletion strains were used to prepare sequencing libraries with the Illumina TruSeq RNA sample preparation Kit. Library preparation started with 2 μg total RNA. After poly-A selection (using poly-T oligo-attached magnetic beads), mRNA was purified and fragmented using divalent cations under elevated temperature. The RNA fragments were reverse transcribed using random primers. A second strand cDNA synthesis was carried out with DNA Polymerase I and RNase H. After end repair and A-tailing, indexing adapters were ligated to the cDNA. The products were then purified and amplified (14 PCR cycles) to create the final cDNA libraries. After library validation and quantification (Agilent 2100 Bioanalyzer), equimolar amounts of library were pooled. The pool was quantified using the Peqlab KAPA Library Quantification Kit and the Applied Biosystems 7900HT Sequence Detection System. The pool was sequenced using an Illumina TruSeq PE Cluster Kit v3 and an Illumina TruSeq SBS Kit v3-HS on an Illumina HiSeq 2000 sequencer with a paired-end (101 x 7 x 101 cycles) protocol.

Sequence reads were mapped to *U*. *maydis* protein encoding genes (ftp://ftpmips.gsf.de/fungi/Ustilaginaceae/Ustilago_maydis_521/) using CLC Genomics Workbench 7.5 (CLC bio). The unique gene reads for all of the 6970 annotated U. maydis genes from the 6 libraries were combined and analyzed in R using the Differentially Expressed Genes (DEG) algorithm edgeR [86]. Differentially expressed genes between FB1 x FB2 and FB1Δros1 x FB2Δros1 were selected on the basis of their fold change (FC ≥ 1.5) and *p*-value (< 0.01).

Expression data were submitted to GeneExpressionOmnibus (http://www.ncbi.nlm.nih.gov/geo/) under the accession number GSE76231.

### ChIP-sequencing

7-day-old maize seedlings of the variety Gaspé Flint (originally provided by B. Burr, Brookhaven National Laboratories) were infected with mixtures of *U*. *maydis* strains FB1Δros1-Ros1HA x FB2Δros1-Ros1HA expressing an HA tagged Ros1 protein or FB1Δros1-Ros1 x FB2Δros1-Ros1 expressing a non-tagged Ros1 protein as negative control. Leaf samples (from 5 different plants) were collected at 8 dpi and incubated in fixation buffer (50 mM HEPES pH 7.5, 1% formaldehyde) for 10 min under vacuum. Excess formaldehyde was quenched by addition of 2 M glycine. Samples were then ground to a fine powder in liquid nitrogen. For chromatin preparation, powder was resuspended in lysis buffer (50 mM HEPES pH 7.5, 150 mM NaCl, 1 mM EDTA, 1% Triton X-100, 5 mM benzamidine, 2 mM PMSF, 1 X Roche complete EDTA free protease inhibitors) and further treated by sonication to lyse the remaining cells using a microtip sonifier (Branson). Chromatin was then sheared in a bioruptor sonication bath (Diagenode) for 10 cycles (30 s on / 30 s off) at high power setting. An aliquot was saved to serve as input control and the rest of the chromatin solution was incubated with ChIP grade Protein A/G magnetic beads (Life technologies) coupled to a monoclonal anti-HA antibody (Sigma) for 10 h at 4°C. Beads were washed three times in lysis buffer, two times in high salt buffer (50 mM HEPES pH 7.5, 300 mM NaCl, 1 mM EDTA, 1% Triton X-100, 1 X Roche complete EDTA free protease inhibitors) and once in TE buffer (10 mM Tris-HCl, 1 mM EDTA, pH 7.5). DNA / Ros1HA complexes were eluted from the beads in TE SDS buffer (10 mM Tris-HCl, 1 mM EDTA, 1% SDS, pH 7.5) by incubation for 15 min at 65°C. Samples and input controls were de-crosslinked for 8–10 h at 65°C in TE SDS buffer containing 200 mM NaCl and 0.65 μg/μL proteinase K. DNA was purified using the ChIP DNA clean and concentrator kit (Zymo research). The experiment was done in three biological replicates which were sequenced separately.

DNA libraries were prepared using the Illumina TruSeq RNA sample preparation Kit v2 starting from the end repair step of the protocol. Up to 100 ng ChIP DNA was used as starting material. After end repair and A-tailing, indexing adapters were ligated. The products were then purified and amplified for 18 PCR cycles to create the final libraries. After validation (Agilent 2200 TapeStation) and quantification using the KAPA Library Quantification Kit (VWR) and the Applied Biosystems 7900HT Sequence Detection System, equimolar amounts of library were pooled. The pool was sequenced using the Illumina TruSeq PE Cluster Kit v3 and the Illumina TruSeq SBS Kit v3-HS (101 x 7 x 101 Cycles) on an Illumina HiSeq 2000 sequencer with a paired-end (101 x 7 x 101 cycles) protocol.

Sequencing data were mapped to *U*. *maydis* genome (ftp://ftpmips.gsf.de/fungi/Ustilaginaceae/Ustilago_maydis_521/) and analyzed using the ChIP-seq tool of the CLC genomics workbench 7.5 software (CLC bio). ChIP-peak discovery was based on read coverage enrichment (*p*-value) and shape of read distribution (peak shape score). ChIP-seq data were submitted to GeneExpressionOmnibus (http://www.ncbi.nlm.nih.gov/geo/) under the accession number GSE76231.

### Quantitative real time and fungal biomass analysis

Gene expression analysis from infected plant material was performed as described in Brefort et al. (2014) [87], with some modifications. Briefly, samples of *U*. *maydis* cells grown in YEPSL and of infected tissue were ground to powder on liquid nitrogen and RNA was extracted with TRIzol (Life technologies). After extraction, the first-strand cDNA synthesis kit (Life technologies) was used to reverse transcribe 1–2 μg of total RNA with oligo(dT) Primers. The qPCR analysis was performed using the SYBR Green Supermix (Life technologies) and an iCycler (Bio-Rad). Cycling conditions were 2 min 95°C, followed by 45 cycles of 30 s 95°C/30 s 62°C/30 s 72°C. The experiment was done in three biological and three technical replicates and gene expression levels were calculated relative to the expression levels of the constitutively expressed fungal gene encoding peptidyl prolyl isomerase (*ppi*). Primers used were ppi_qF / R for the reference gene *ppi* and primer pairs ros1_qF / R, rum1_qF / qR, ust1_qF / qR, hgl1_qF / qR, tup1_qF / qR, 05550_qF / qR, 04503_qF / qR, 02212_qF / qR, 01070_qf/qR, pks1_qF / qR, 04101_qF/qR, biz1_qF / qR, rbf1_qF / qR, fox1_qF / qR, mig2-3_qF / qR, 04096_qF / qR, dik1_qF / qR, 02473_qF / qR, and 03046_qF / qR for *ros1*, *rum1*, *ust1*, *hgl1*, *tup1*, *UMAG_05550*, *UMAG_04503*, *UMAG_02212*, *UMAG_01070*, *pks1*, *UMAG_04101*, *biz1*, *rbf1*, *fox1*, *mig2-3*, *UMAG_04096*, *dik1*, *UMAG_02473*, *UMAG_03046* respectively. All primer sequences are listed in S6 Table. Relative expression was determined using the ΔΔCt method [88]. *t*-tests were used to assess statistically relevant differences between expression levels at different time points (*p* ≤ 0.05).

Quantification of relative fungal biomass in infected maize leaves was performed as described previously [87], with some modifications. 2 cm long sections from 10 leaves with the most prominent symptoms were harvested from 10 different plants at the indicated time points. For genomic DNA extraction leaf material was frozen in liquid nitrogen, ground to a fine powder, and extracted using a phenol-based protocol modified from Hoffman and Winston (1987) [89]. The qPCR analysis was performed using the Platinum SYBR Green Supermix (Life technologies) in an iCycler (Bio-Rad). Cycling conditions were 2 min 95°C, followed by 45 cycles of 30 s 95°C / 30 s 62°C / 30 s 72°C. *U*. *maydis* biomass was quantified with primers ppi_qF/R amplifying the fungal *ppi* gene. Maize glyceraldehyde 3-phosphate dehydrogenase was amplified with primers Gapdh_qF/R and served as reference gene for normalization. The experiment was done in three biological and three technical replicates. *t*-tests were used to assess statistically relevant differences among strains (*p* ≤ 0.05).

### Staining methods for microscopy

Wheat germ agglutinin-Alexa Fluor 488 / propidium iodide staining of infected leaf material was performed as described previously [38]. For staining of the mucilaginous matrix, leaf tumors from maize seedlings infected with strains FB1 x FB2 or FB1∆ros1 x FB2∆ros1 were collected at 10 dpi. Samples were fixed in 4% glutaraldehyde and embedded in Epoxy resin. 1–2 μm thick sections were generated with a microtome and stained with methylene blue-azure II-basic fuchsin following the protocol described by Humphrey and Pittman (1974) [49].

### Confocal imaging

To examine fungal colonization of leaf tissue, samples from infected plants were fixed in ethanol, transferred to 10% KOH, incubated at 85°C for 4 hours, washed twice with PBS buffer (140 mM NaCl, 16 mM Na_2_HPO_4_, 2 mM KH_2_PO_4_, 3.5 mM KCl, and 1 mM Na_2_-EDTA, pH 7.4), and incubated under vacuum in staining solution (10 μg/mL propidium iodide and 10 μg/mL WGA Alexa Fluor 488 in PBS, pH 7.4) according to Doehlemann *et al*. (2008) [38]. WGA Alexa Fluor 488 was purchased from Life technologies. To visualize the septa in strains expressing *ros1* in axenic culture, cell walls were stained with calcofluor (Fluorescent brightener 28). Nuclei were stained with 4',6-diamidino-2-phenylindole (DAPI).

For microscopy, an Axioplan II microscope (Zeiss) with differential interference contrast optics was used. Fluorescence of GFP, mCherry, calcofluor and DAPI was observed using GFP (ET470/40BP, ET495LP, and ET525/50BP), rhodamine (HC562/40BP, HC593LP, and HC624/40BP), and DAPI (HC375/11BP, HC409BS, and HC447/60BP) filter sets (Semrock). Pictures were taken with a CoolSNAP-HQ charge-coupled device camera (Photometrics). Image processing was done with MetaMorph software (Universal Imaging).

Confocal microscopy was performed using a TCS-SP5 confocal microscope (Leica Microsystems). GFP and wheat germ agglutinin-Alexa Fluor 488 were excited at 488 nm and emitted fluorescence was detected in the 495–530 nm range. Propidium iodide and mCherry were excited at 561 nm and emission was detected in the 580–630 nm range. Images were processed using LAS-AF software (Leica Microsystems).

### Recombinant protein expression and purification

To allow the recombinant expression of the Ros1WOPR domain comprising amino acids 1–321 of Ros1, this domain was C-terminally fused to a His-tag (Ros1WOPR-His). To this end *ros1* was amplified with primers WOPR_NdeF / WOPR_XhoR, cloned in pET28 (Novagen) to generate pRos1WORP-His and transformed into *E*. *coli* strain BL21(DE3)pLysS (Promega). Expression of Ros1WOPR-His was induced in exponentially growing cell cultures for 4h at 28°C in dYT medium supplemented with 0.15% glucose, 1 mM MgSO_4_ and 0.5 mM IPTG. To achieve cell lysis cell pellets were resuspended in BugBuster Master Mix reagent (Merck Millipore) supplemented with protease inhibitors (complete EDTA-free tablet, Roche) and incubated for 20 min at room temperature. The crude cell extract was then centrifuged (30000 x g; 30 min) and the supernatant was loaded on a Ni-NTA column (HisTrap FF Crude, GE Healthcare) equilibrated in wash buffer (50 mM Na_2_HPO_4_, 300 mM NaCl, 20 mM imidazole, pH 8.0) using an Äkta FPLC system (GE Healthcare). After unbound protein was washed off the Ros1WOPR–His protein was eluted with elution buffer (50 mM Na_2_HPO_4_, 300 mM NaCl, pH 8.0, 135 mM imidazole). The Ros1WOPR-His containing fractions were pooled and loaded on a Superdex75 gel filtration column (SuperdexTM 75 10/300GL, GE Healthcare) equilibrated in gel filtration buffer (20 mM HEPES, 20 mM NaCl, 0.1 μM PMSF and 1 mM DTT). The Ros1WOPR-His containing fractions were pooled and incubated in batch with source 15Q anion exchange beads (GE Healthcare) equilibrated in gel filtration buffer. Ros1WOPR-His remained in the supernatant, which was concentrated via Amicon Ultra-4 centrifugation units with an Ultracel-3 membrane (Merck Millipore). The protein was stored at 4°C for up to five days.

### Electrophoretic Mobility Shift Assays (EMSA)

For *ros1* WT-probe and ORF-probes, pC*ros1* was used as template. For the mutated versions of the *ros1* WT-probe, corresponding fragments were generated by annealing several overlapping oligonucleotides carrying the desired mutations and cloning them into p123 [81] between restriction NdeI and BamHI sites The inserts in the resulting plasmids pm1, pm2 and pm1+2 were sequenced and plasmids were subsequently used as template for the respective PCR reactions using 5’ biotin labeled primers WT-probe_F/R for the WT-probe and the mutated versions m1, m2 and m1+2 and primers ORF-probe_F/R for the ORF_probe. Wild type competitor DNA corresponding to the same sequence as WT-probe was PCR amplified using unlabeled WT-probe_F/R primers. Probes corresponding to promoter regions upstream of *UMAG_02854*, *UMAG_04040*, *UMAG_02538*, *cmu1*, *UMAG_03046*, *UMAG_03138*, *UMAG_12258*, and *UMAG_02775* were generated by PCR using 5’ biotin labeled primer pairs Probe-02854_F / R, Probe-04040_F / R, Probe-02538_F / R, Probe-cmu1_F / R, Probe-03046_F / R, Probe-03138_F / R, Probe-12258_F / R, Probe-02775_F1 / R1 and Probe-02775_F2 / R2 respectively. Competitors were generated by PCR using corresponding non labeled primers. All primer sequences are listed in S6 Table. EMSAs were performed using the Lightshift Chemiluminescent EMSA Kit (Thermofischer). Binding reactions were carried out in 20 mM HEPES buffer, pH 8.0 supplemented with 1 mM DTT, 50 mM NaCl, 25 ng/μL poly dI/dC, 2.5% glycerol 0.05% NP40, 5 mM MgCl2 and 1 μg/μL BSA. 10 fmol of biotin labeled dsDNA probe and 3 μg purified Ros1WOPR-His protein were used per binding reaction. For competition reactions, competitor fragment was added in a 500-fold molar excess. Binding reactions were incubated for 30 min at room temperature. In competition experiments Ros1WOPR was incubated for 20 min with the non-labeled competitor prior to addition of the probe. Binding reactions were separated on native 4% polyacrylamide 0.5 X TBE gels. Gels were transferred to a nylon membrane and biotin-labeled DNA fragments were detected using a streptavidin horseradish peroxidase conjugate and a highly sensitive chemiluminescent substrate as recommended by the manufacturer (Thermofischer).

### Accession numbers

ros1 (*UMAG_05853*): XM_011393913; *UMAG_02775*: XM_011390829; *UMAG_01390*: XM_011388973

## Supporting Information

S1 Fig Ros1 belongs to the WOPR family of transcriptional regulators. (A) Schematic representation of the domain structure of *U*. *maydis* Ros1 protein (UmRos1, XP_011392215) and other members of the WOPR family including all WOPR proteins which have been experimentally characterized: *Zymoseptoria tritici* Wor1 (ZmWor1, AHH91582), *Cladosporium fulvum* Wor1 (CfWor1, JGI ID: 183744), *Fusarium verticillioides* Sge1 (FvSge1, W7MPI5), *Fusarium oxysporum f*. *sp*. *Lycopersici* Sge1 (FoSge1, AGA55574), *Verticillium dahlia* Sge1 (VdSge1, EGY16897), *Magnaporthe oryzae* GTI1 (MoGTI1, ELQ65940), *Fusarium graminearum* Fgp1 (FgFgp1, I1S5P3), *Botrytis cinerea* Reg1 (BcReg1, XP_001546736), *Candida albicans* Wor1 (CaWor1, Q5AP80), *Saccharomyces cerevisiae* Mit1 (ScMit1, P40002), *Schizosaccharomyces pombe* Gti1 (SpGti1, CAB61447). Also included are the two WOPR proteins showing the highest similarity to Ros1, SrWopr from *Sporisorium reilianum* (CBQ70896) and CnWopr from *Cryptococcus neoformans* (KIR63833). The WOPR proteins from basidiomycetes, SrWopr, UmRos1 and CnWopr, are indicated in blue. The two conserved domains forming the WOPR box, WOPRa and WOPRb are indicated in red boxes with a lighter shade for WOPRb. The blue triangles represent predicted nuclear localization signals (NLS) and the green ellipses represent Glutamine-rich regions. The top scale bar indicates the size in bp. (B) Alignment of the amino acid sequences of the WOPRa and WOPRb segments from Ros1 and its orthologues. The 15 amino acid residues involved in the interaction of Wor1 with DNA are indicated by stars. The recognition loop (R loop) which recognizes the core DNA motif in Wor1 is indicated by a purple line.
(TIF)

S2 Fig *ros1* mutants are able to mate and filament. Strains FB1, FB2, FB1Δros1 and FB2Δros1 were grown in YEPSL to an OD_600_ of 1.0, washed and resuspended in water. The strains indicated on top were spotted alone and in combinations with the strains indicated on the left side on charcoal-containing PD plates and incubated at room temperature for 48h. White fuzziness indicating the presence of dikaryotic filaments was visible for all strain combinations including FB1Δros1 x FB2Δros1 indicating that mating is not affected by the deletion of *ros1*.
(TIF)

S3 Fig Validation of putative Ros1 targets identified by RNA-seq results through RT-qPCR. The expression of genes encoding glycoside hydrolases (*UMAG_05550*, *UMAG_04503*), a trehalase: (*UMAG_02212*), a cyclopropane fatty acid synthase (*UMAG_01070*), a polyketide synthase (*pks1*), transcription factors (*UMAG_04101*, *biz1*, *rbf1*, *fox1*, *UMAG_02775*) and secreted effectors (*UMAG_04096*, *dik1*, *UMAG_02473*, *UMAG_03046*) was determined by qRT-PCR for the wild type strains FB1 x FB2 (grey bars) and the corresponding *ros1* deletion strains (white bars) 8 days after infection of maize seedlings. The constitutively expressed *ppi* gene (*UMAG_03726*) was used for normalization. Relative expression was determined using the ΔΔCt method. Values shown are means of three biological replicates. Bars indicate the standard deviation between biological replicates. All differences observed between strains are statistically significant (unpaired *t*-test, *p* ≤ 0.05).
(TIF)

S4 Fig Ros1 is involved in the regulation of *rum1*, *ust1 and hgl1*. qRT-PCR analysis of *rum1*, *ust1*, *hgl1 and tup1* expression during plant infection by the wild type strains FB1 x FB2 or the corresponding *ros1* deletion strains. Infected plant samples were collected at the time-points indicated below. qRT-PCR analysis was performed using the constitutively expressed *ppi* gene (*UMAG_03726*) for normalization. Relative expression was determined using the ΔΔCt method. Values shown are means of three biological replicates. Bars indicate the standard deviation between biological replicates. Asterisks indicate significant differences between strains (unpaired *t*-test, *p* ≤ 0.05). While *rum1*, *hgl1* and *ust1* are slightly induced by Ros1 at late time points, *tup1* expression is not differentially regulated by Ros1.
(TIF)

S5 Fig In vitro binding of Ros1WOPR-His to several target promoters identified by ChIP. Ros1WOPR-His expressed and purified from *E*. *coli* was used in EMSA assays with probes corresponding to target sequences identified by ChIP in the promoters of the transcription factor gene *UMAG_02775* (A), the downregulated effector genes *UMAG_02854*, *UMAG_04040*, *UMAG_02538* and *cmu1* (B) and the upregulated effector genes *UMAG_03138*, *UMAG_12258* and *UMAG_03046* (C). Probes were amplified by PCR with primers listed in S6 Table, each probe is predicted to contain at least one binding sites for Ros1. When incubated with Ros1WOPR-His, all specific probes were shifted and this could be competed by addition of the corresponding specific non-labeled probe as competitor. The different complex mobilities observed for different probes bound by Ros1 most likely reflect different topologies of the Ros1WOPR-His–DNA complex. As negative control not bound by Ros1, an ORF-probe corresponding to a part of the *ros1* coding sequence was included on the different gels.
(TIF)

S6 Fig Ros1 induces the expression of the transcription factor genes *UMAG_01390* and *UMAG_02775* late during infection. qRT-PCR analysis of *UMAG_02775 and UMAG_01390* expression during plant infection by the wild type strains FB1 x FB2 (grey bars) or the corresponding *ros1* deletion strains (white bars). Infected plant samples were collected at the time-points indicated below. qRT-PCR analysis was performed using the constitutively expressed *ppi* gene (*UMAG_03726*) for normalization. Relative expression was determined using the ΔΔCt method. Values shown are means of three biological replicates. Bars indicate the standard deviation between biological replicates. Asterisks indicate significant differences between strains (unpaired *t*-test, *p* ≤ 0.05).
(TIF)

S7 Fig Virulence of mutants lacking either *UMAG_02775* or *UMAG_01390*. Wild type strains FB1 and FB2 and the corresponding *UMAG_02775 and UMAG_01390* deletion and complementation strains were mixed in the indicated combinations and injected into maize seedlings. Disease symptoms were scored 12 days after infection according to Kämper et al. (2006) [10]. Colors used for disease scores are indicated on the right side. Three independent experiments were performed and the average values are expressed as a percentage of the total number of infected plants (n) given above each column. *UMAG_02775* deletion strains do not show any virulence defect while *UMAG_01390* deletion strains show a similar virulence phenotype as the *ros1* deletion strains.
(TIF)

S1 Table *ros1*-dependently regulated *U*. *maydis* genes 8 days after infection determined by RNA-seq. (XLSX)

S2 Table List of genomic regions bound by Ros1 determined by ChIP-seq analysis. (XLSX)

S3 Table List of potential direct Ros1 targets in *U*. *maydis*. (XLSX)

S4 Table Occurence of motifs related to the Wor1 binding site in the ChIP peak sequences. (XLSX)

S5 Table *U*. *maydis* strain list. (DOCX)

S6 Table Primer sequences. (DOCX)
