# Supplementary figures and images for: The WOPR Protein Ros1 Is a Master Regulator of Sporogenesis and Late Effector Gene Expression in the Maize Pathogen Ustilago maydis
Source: PLoS Pathog. 2016 Jun 22;12(6):e1005697. doi: 10.1371/journal.ppat.1005697 (PMC4917244; doi:10.1371/journal.ppat.1005697)

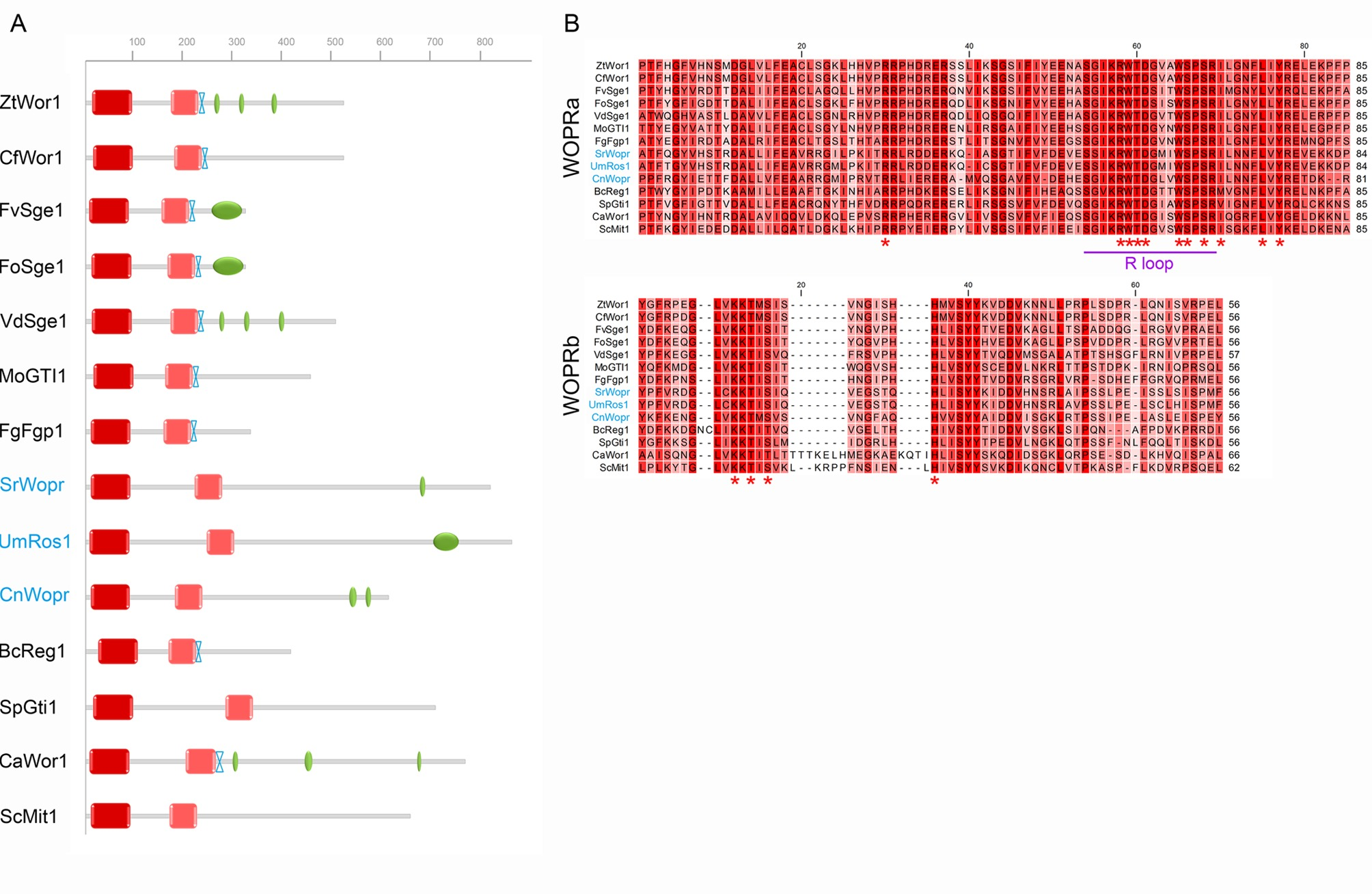

Supplement: S1 Fig — (A) Schematic representation of the domain structure of U. maydis Ros1 protein (UmRos1, XP_011392215) and other members of the WOPR family including all WOPR proteins which have been experimentally characterized: Zymoseptoria tritici Wor1 (ZmWor1, AHH91582), Cladosporium fulvum Wor1 (CfWor1, JGI ID: 183744), Fusarium verticillioides Sge1 (FvSge1, W7MPI5), Fusarium oxysporum f. sp. Lycopersici Sge1 (FoSge1, AGA55574), Verticillium dahlia Sge1 (VdSge1, EGY16897), Magnaporthe oryzae GTI1 (MoGTI1, ELQ65940), Fusarium graminearum Fgp1 (FgFgp1, I1S5P3), Botrytis cinerea Reg1 (BcReg1, XP_001546736), Candida albicans Wor1 (CaWor1, Q5AP80), Saccharomyces cerevisiae Mit1 (ScMit1, P40002), Schizosaccharomyces pombe Gti1 (SpGti1, CAB61447). Also included are the two WOPR proteins showing the highest similarity to Ros1, SrWopr from Sporisorium reilianum (CBQ70896) and CnWopr from Cryptococcus neoformans (KIR63833). The WOPR proteins from basidiomycetes, SrWopr, UmRos1 and CnWopr, are indicated in blue. The two conserved domains forming the WOPR box, WOPRa and WOPRb are indicated in red boxes with a lighter shade for WOPRb. The blue triangles represent predicted nuclear localization signals (NLS) and the green ellipses represent Glutamine-rich regions. The top scale bar indicates the size in bp. (B) Alignment of the amino acid sequences of the WOPRa and WOPRb segments from Ros1 and its orthologues. The 15 amino acid residues involved in the interaction of Wor1 with DNA are indicated by stars. The recognition loop (R loop) which recognizes the core DNA motif in Wor1 is indicated by a purple line. (TIF) [file ppat.1005697.s001.tif]

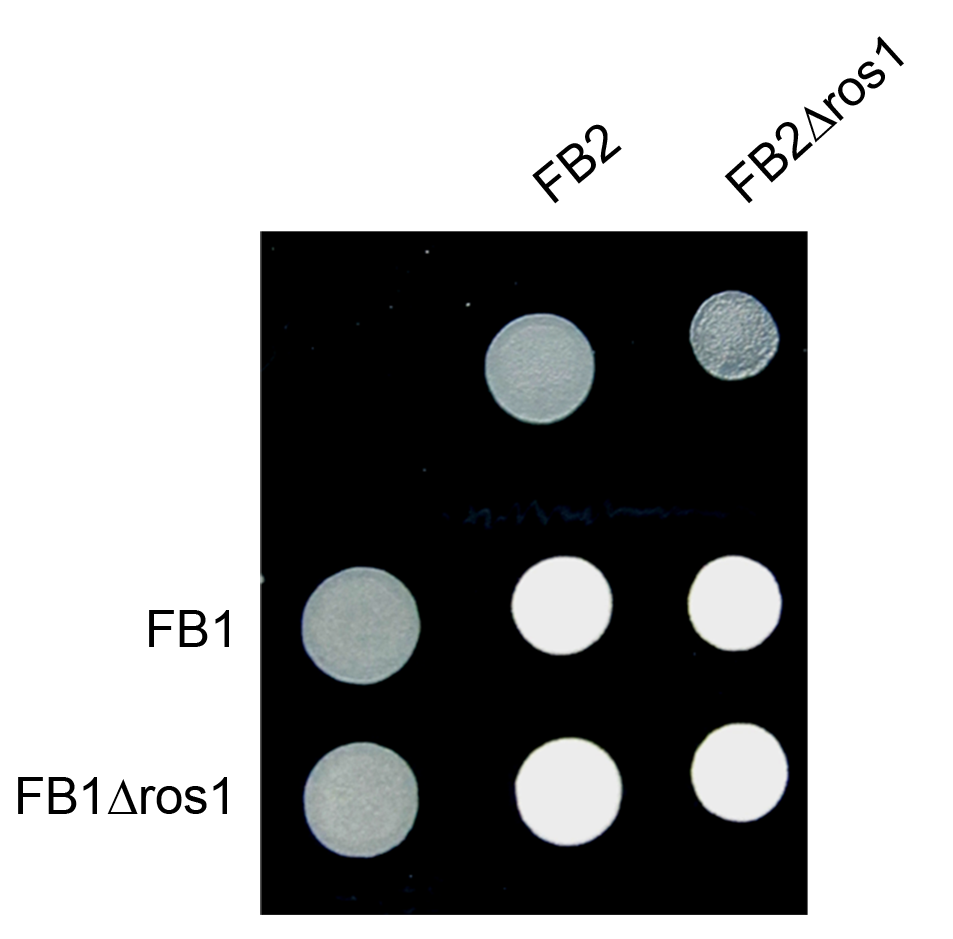

Supplement: S2 Fig — Strains FB1, FB2, FB1Δros1 and FB2Δros1 were grown in YEPSL to an OD600 of 1.0, washed and resuspended in water. The strains indicated on top were spotted alone and in combinations with the strains indicated on the left side on charcoal-containing PD plates and incubated at room temperature for 48h. White fuzziness indicating the presence of dikaryotic filaments was visible for all strain combinations including FB1Δros1 x FB2Δros1 indicating that mating is not affected by the deletion of ros1. (TIF) [file ppat.1005697.s002.tif]

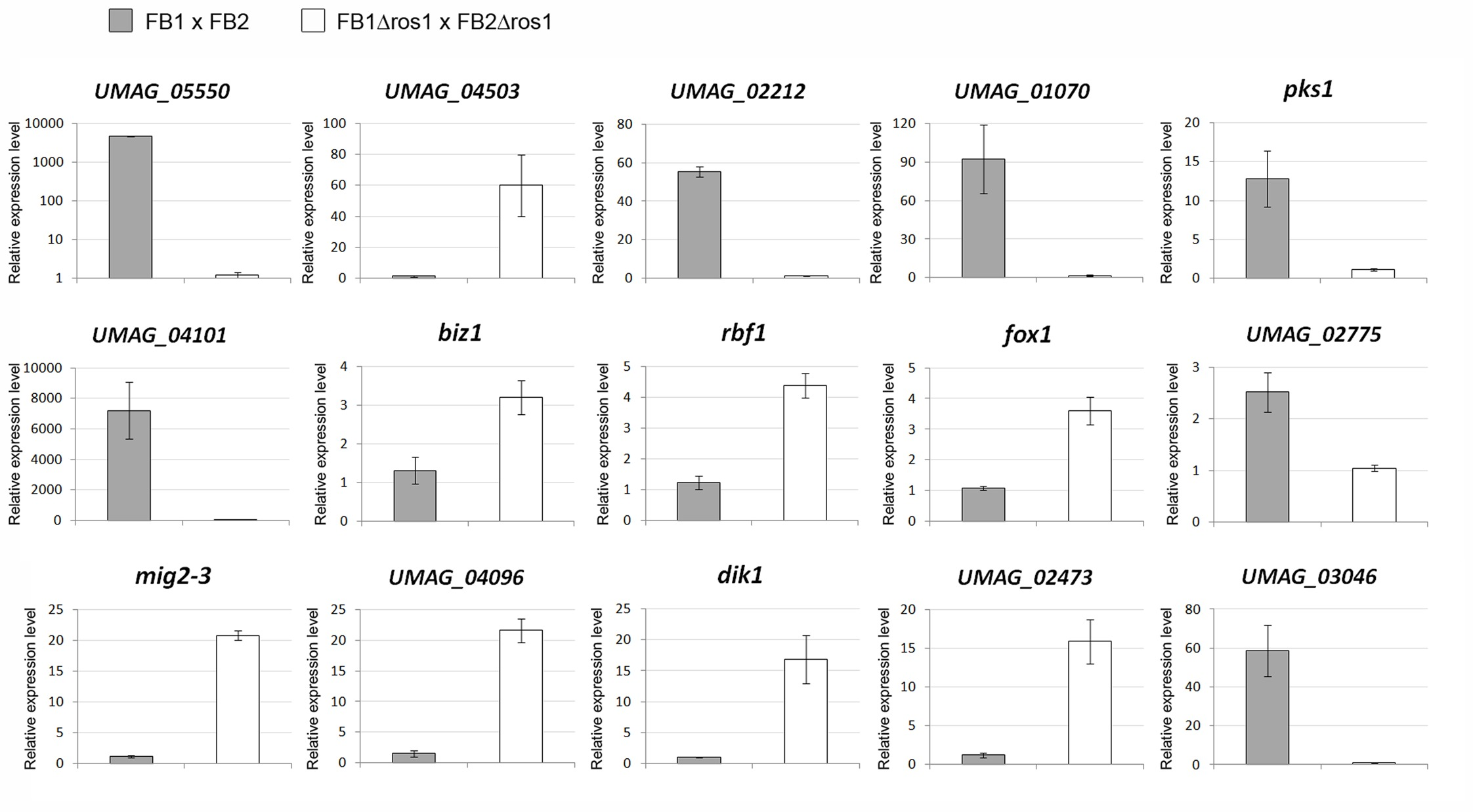

Supplement: S3 Fig — The expression of genes encoding glycoside hydrolases (UMAG_05550, UMAG_04503), a trehalase: (UMAG_02212), a cyclopropane fatty acid synthase (UMAG_01070), a polyketide synthase (pks1), transcription factors (UMAG_04101, biz1, rbf1, fox1, UMAG_02775) and secreted effectors (UMAG_04096, dik1, UMAG_02473, UMAG_03046) was determined by qRT-PCR for the wild type strains FB1 x FB2 (grey bars) and the corresponding ros1 deletion strains (white bars) 8 days after infection of maize seedlings. The constitutively expressed ppi gene (UMAG_03726) was used for normalization. Relative expression was determined using the ΔΔCt method. Values shown are means of three biological replicates. Bars indicate the standard deviation between biological replicates. All differences observed between strains are statistically significant (unpaired t-test, p ≤ 0.05). (TIF) [file ppat.1005697.s003.tif]

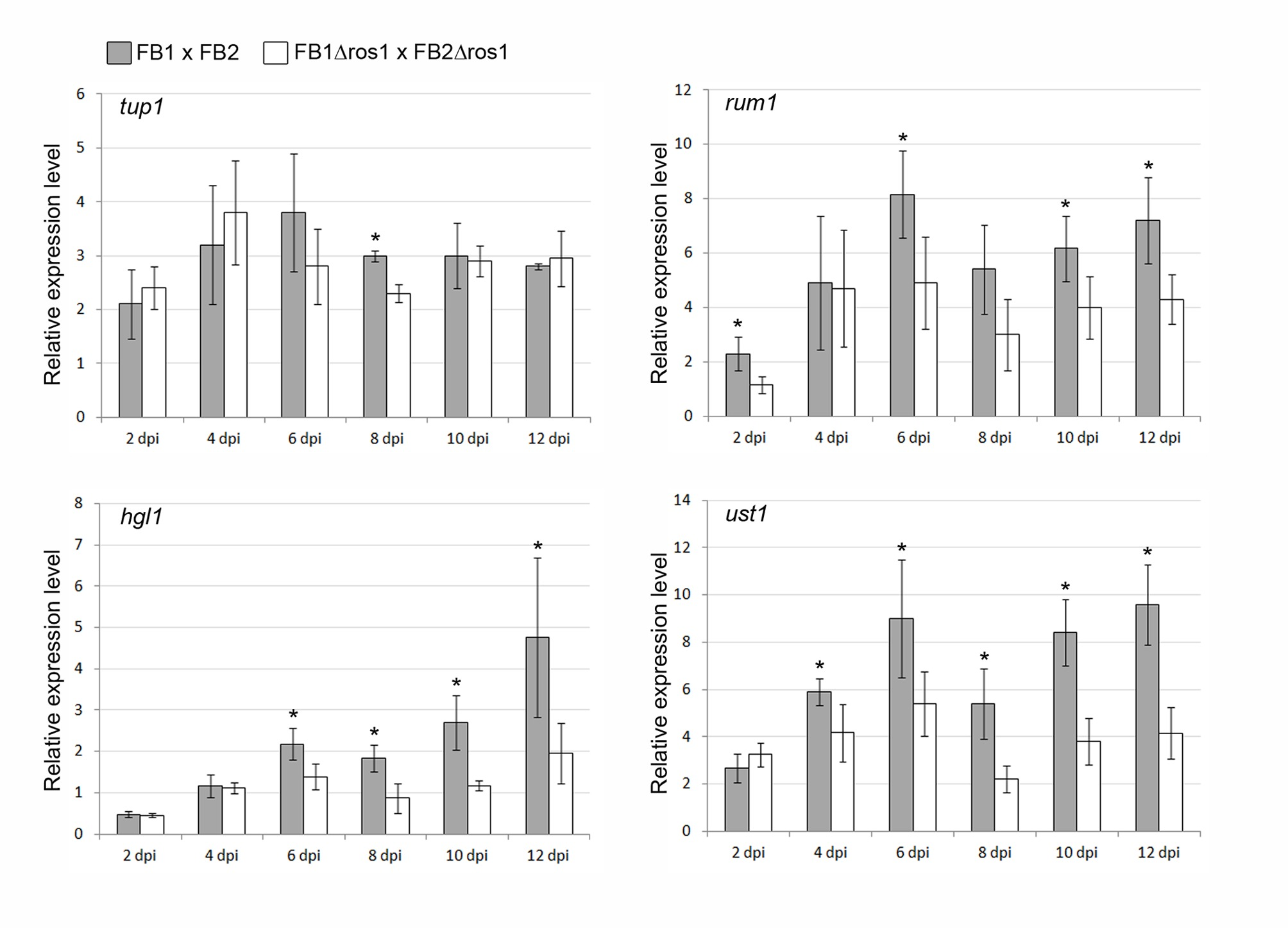

Supplement: S4 Fig — qRT-PCR analysis of rum1, ust1, hgl1 and tup1 expression during plant infection by the wild type strains FB1 x FB2 or the corresponding ros1 deletion strains. Infected plant samples were collected at the time-points indicated below. qRT-PCR analysis was performed using the constitutively expressed ppi gene (UMAG_03726) for normalization. Relative expression was determined using the ΔΔCt method. Values shown are means of three biological replicates. Bars indicate the standard deviation between biological replicates. Asterisks indicate significant differences between strains (unpaired t-test, p ≤ 0.05). While rum1, hgl1 and ust1 are slightly induced by Ros1 at late time points, tup1 expression is not differentially regulated by Ros1. (TIF) [file ppat.1005697.s004.tif]

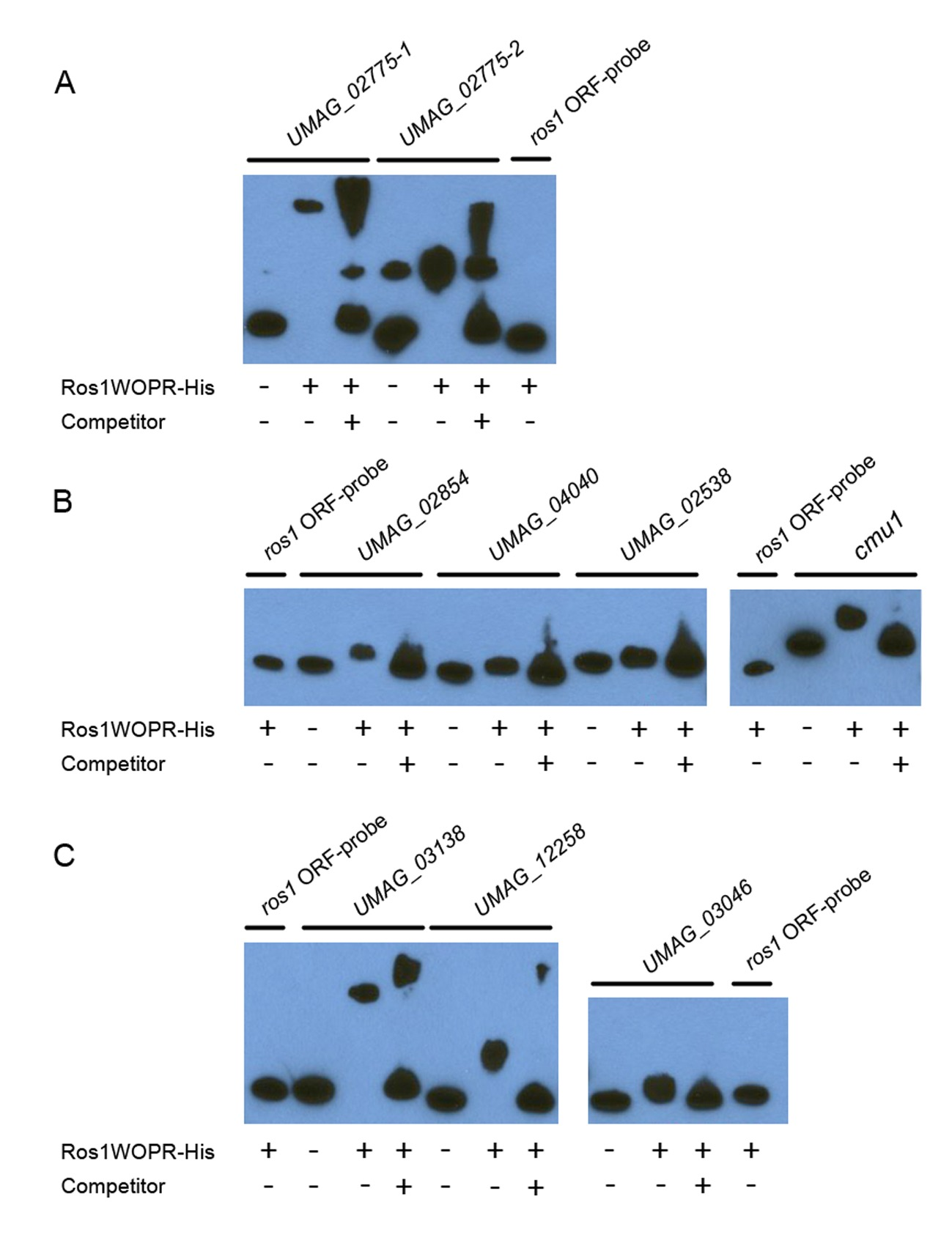

Supplement: S5 Fig — Ros1WOPR-His expressed and purified from E. coli was used in EMSA assays with probes corresponding to target sequences identified by ChIP in the promoters of the transcription factor gene UMAG_02775 (A), the downregulated effector genes UMAG_02854, UMAG_04040, UMAG_02538 and cmu1 (B) and the upregulated effector genes UMAG_03138, UMAG_12258 and UMAG_03046 (C). Probes were amplified by PCR with primers listed in S6 Table, each probe is predicted to contain at least one binding sites for Ros1. When incubated with Ros1WOPR-His, all specific probes were shifted and this could be competed by addition of the corresponding specific non-labeled probe as competitor. The different complex mobilities observed for different probes bound by Ros1 most likely reflect different topologies of the Ros1WOPR-His–DNA complex. As negative control not bound by Ros1, an ORF-probe corresponding to a part of the ros1 coding sequence was included on the different gels. (TIF) [file ppat.1005697.s005.tif]

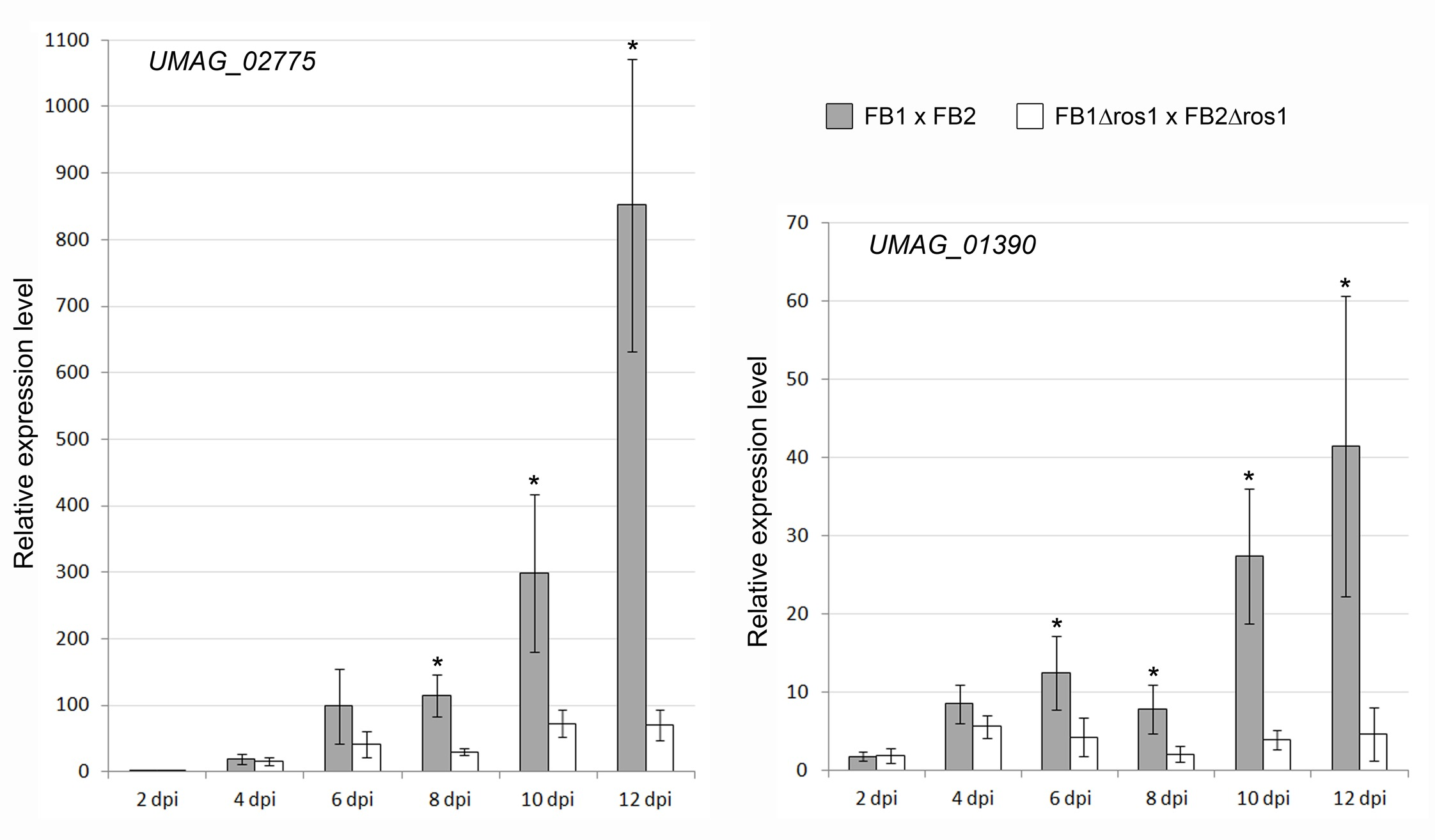

Supplement: S6 Fig — qRT-PCR analysis of UMAG_02775 and UMAG_01390 expression during plant infection by the wild type strains FB1 x FB2 (grey bars) or the corresponding ros1 deletion strains (white bars). Infected plant samples were collected at the time-points indicated below. qRT-PCR analysis was performed using the constitutively expressed ppi gene (UMAG_03726) for normalization. Relative expression was determined using the ΔΔCt method. Values shown are means of three biological replicates. Bars indicate the standard deviation between biological replicates. Asterisks indicate significant differences between strains (unpaired t-test, p ≤ 0.05). (TIF) [file ppat.1005697.s006.tif]

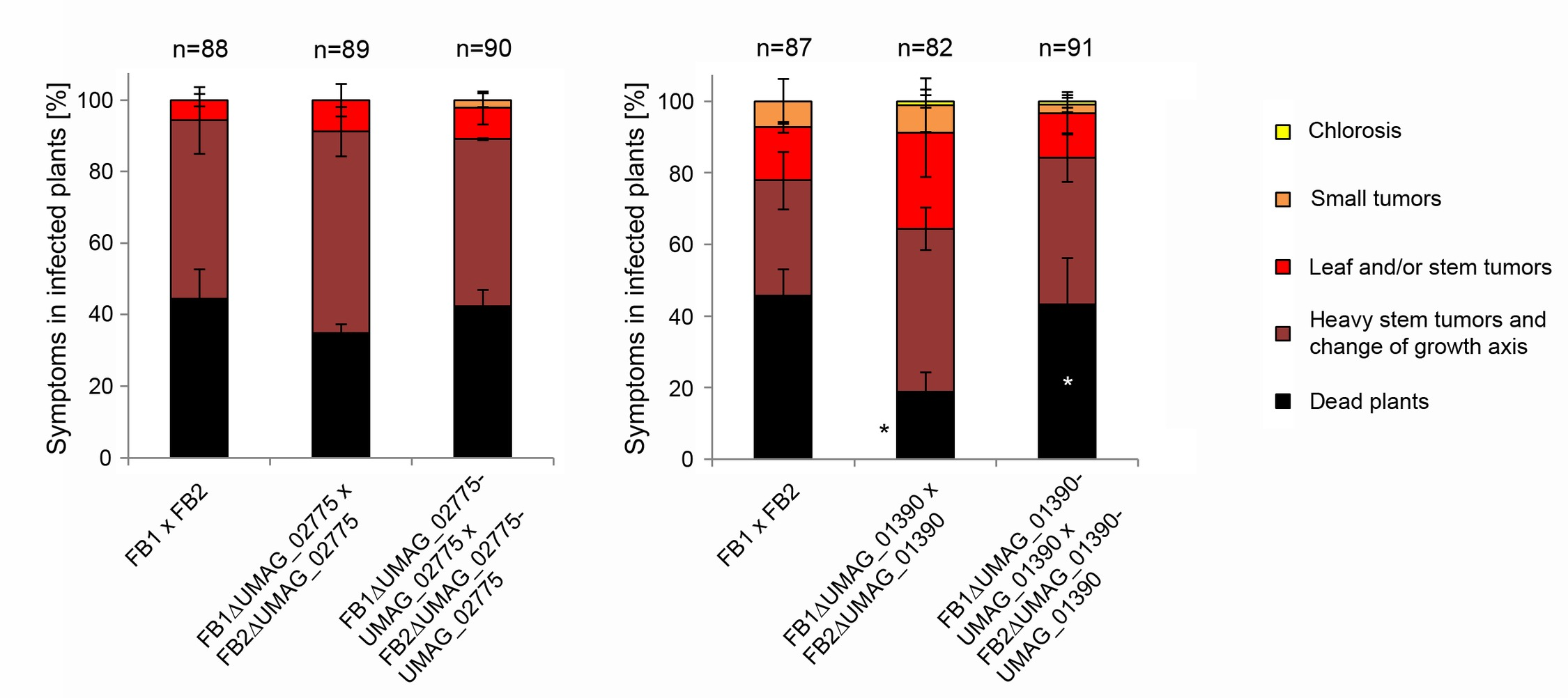

Supplement: S7 Fig — Wild type strains FB1 and FB2 and the corresponding UMAG_02775 and UMAG_01390 deletion and complementation strains were mixed in the indicated combinations and injected into maize seedlings. Disease symptoms were scored 12 days after infection according to Kämper et al. (2006) [10]. Colors used for disease scores are indicated on the right side. Three independent experiments were performed and the average values are expressed as a percentage of the total number of infected plants (n) given above each column. UMAG_02775 deletion strains do not show any virulence defect while UMAG_01390 deletion strains show a similar virulence phenotype as the ros1 deletion strains. (TIF) [file ppat.1005697.s007.tif]
