# Supplementary material for: The WOPR Protein Ros1 Is a Master Regulator of Sporogenesis and Late Effector Gene Expression in the Maize Pathogen Ustilago maydis
Source: PLoS Pathog. 2016 Jun 22;12(6):e1005697. doi: 10.1371/journal.ppat.1005697 (PMC4917244; doi:10.1371/journal.ppat.1005697)
Supplement: S5 Table — (DOCX) [file ppat.1005697.s012.docx]

**Table S5:** *U. maydis* strain list

| Strain | Relevant genotype | Resistance^1^ | Reference |
| --- | --- | --- | --- |
| FB1 | *a1b1* |  | Banuett & Herskowitz, 1989 |
| FB2 | *a2b2* |  | Banuett & Herskowitz, 1989 |
| FB1P_otef_Ros1mCherry-P_nup107_Nup107eGFP | *a1b1 nup107:egfp ble ip^r^[P_otef_:um05853:mcherry]ip^s^* | P, C | This study |
| FB1Δros1 | *a1b1*; *ros1*::*hyg* | H | This study |
| FB2Δros1 | *a2b2*; *ros1*::*hyg* | H | This study |
| FB1Δros1-Ros1 | *a1b1* *ros1*::*hyg* *ip^r^*[P*_ros1_*:*ros1*]*ip^s^* | H, C | This study |
| FB1Δros1-Ros1 | *a2b2* *ros1*::*hyg* *ip^r^*[P*_ros1_*:*ros1*]*ip^s^* | H, C | This study |
| FB1_Pnup107_Nup107eGFP-_Psso1_Sso1mCherry | *a1b1* *nup107*:*egfp* *ble* *ip^r^*[P*_sso1_*:*sso1*:*mcherry*]*ip^s^* | P, C | This study |
| FB2_Pnup107_Nup107eGFP- _Psso1_Sso1mCherry | *a2b2* *nup107*:*egfp* *ble* *ip^r^*[P*_sso1_*:*sso1*:*mcherry*]*ip^s^* | P, C | This study |
| FB1Δros1_Pnup107_Nup107eGFP- _Psso1_Sso1mCherry | *a1b1 ros1::hyg nup107:egfp ble ip^r^[P_sso1_:sso1:mcherry]ip^s^* | P, C, H | This study |
| FB2Δros1_Pnup107_Nup107eGFP- _Psso1_Sso1-mCherry | *a2b2 ros1::hyg nup107:egfp ble ip^r^[P_sso1_:sso1:mcherry]ip^s^* | P, C, H | This study |
| FB1Δros1_Pmig2-6_Ros1 | *a1b1, ros1::hyg ip^r^[P_mig2.6_:ros1]ip^s^* | H, C | This study |
| FB2Δros1_Pmig2-6_Ros1 | *a2b2, ros1::hyg ip^r^[P_mig2.6_:ros1]ip^s^* | H, C | This study |
| AB33 | *a2 P_nar_:bW2 P_nar_:bE1 ble* | P | Brachmann et al, 2001 |
| AB33_Pcrg1_Ros1 | *a2 P_nar_:bW2 P_nar_:bE1 ble P_crg1_:ros1 cbx* | P, C | This study |
| FB1Δros1-Ros1HA | *a1b1 ros1:hyg ip^r^[P_ros1_:ros1:3xHA]ip^s^* | H, C | This study |
| FB2Δros1-Ros1HA | *a2b2 ros1:hyg ip^r^[P_ros1_:ros1:3xHA]ip^s^* | H, C | This study |
| FB1ΔUMAG_02775 | *a1b1, um02775::hyg* | H | This study |
| FB2ΔUMAG_02775 | *a2b2, um02775::hyg* | H | This study |
| FB1ΔUMAG_02775-UMAG_02775 | *a1b1, um02775::hyg ipr[Pum02775:um02775]ips* | H, C | This study |
| FB2ΔUMAG_02775-UMAG_02775 | *a2b2, um02775::hyg ipr[Pum02775:um02775]ips* | H, C | This study |
| FB1ΔUMAG_01390 | *a1b1, um01390::hyg* | H | This study |
| FB2ΔUMAG_01390 | *a2b2, um01390::hyg* | H | This study |
| FB1ΔUMAG_01390-UMAG_01390 | *a1b1, um01390::hyg ipr[Pum01390:um01390]ips* | H, C | This study |
| FB2ΔUMAG_01390-UMAG_01390 | *a2b2, um01390::hyg ipr[Pum01390:um01390]ips* | H, C | This study |

^1^ Hygromycin resistance (H), Carboxin resistance (C), Phleomycin resistance (P)
