# Supplementary material for: The WOPR Protein Ros1 Is a Master Regulator of Sporogenesis and Late Effector Gene Expression in the Maize Pathogen Ustilago maydis
Source: PLoS Pathog. 2016 Jun 22;12(6):e1005697. doi: 10.1371/journal.ppat.1005697 (PMC4917244; doi:10.1371/journal.ppat.1005697)
Supplement: S6 Table — (DOCX) [file ppat.1005697.s013.docx]

**Table S6:** Primer list

| Primer | Sequence 5’-3’ |
| --- | --- |
| Ros1-mCherry-HA fusion | |
| mcherry_NotR | CTATGCGGCCGCTTAAGCGTAATCTGGAACATCGT |
| mcherry_EcoF | CTATGAATTCAAGGGCGAGGAGGATAACATGG |
| ros1_XmaF | CTATCCCGGGATGGGTTCACACCAAGCTGCTA |
| ros1_EcoR | CTATGAATTCCGGCACAGGTCTCGTCAACAC |
| *ros1* deletion | |
| Dros1LB_F | GTGGAAGCTTGCTGGTCCGATCTTCATTTAGCGT |
| Dros1LB_R | CAGGCCTGAGTGGCCAGGGATTGGGCCTGAAACAGAAAG |
| Dros1RB_F | CAGGCCATCTAGGCCGTGACCGTCCCAATCTGTACTAAC |
| Dros1RB_R | GTGTCTAGATCACGACTGACAACTGTGACAAGC |
| Dros1_F | TCTGCATTCGATTCCACCTTGCTC |
| Dros1_R | GCTTGGCAGGCAATCACAGAAT |
| *UMAG_02775* deletion | |
| D02775LB_F | GTAACGCCAGGGTTTTCCCAGTCACGACGCCTTAGGAAGATTGTCCCGTCTGTCTCGAAAC |
| D02775LB_R | CGATGATAAGCTGTCAAACATGAGGCCTGAGTGGCCTCGAGGGCGTTTCAAGCTGAATCCG |
| D02775RB_F | ACTCGCTGGTAGTTACCACGTTCGGCCATCTAGGCCCCATCTGCAACGCGACACGAGACT |
| D02775RB_R | GCGGATAACAATTTCACACAGGAAACAGCCCTAAGGACTGGTGCTGTTCACGCTGCTGCTCA |
| *UMAG_01390 deletion* | |
| D01390LB_F | GTAACGCCAGGGTTTTCCCAGTCACGACGCCTTAGGCACCAACTTAGCTCCTTCCGCTTT |
| D01390LB_R | CGATGATAAGCTGTCAAACATGAGGCCTGAGTGGCCAAGACGAGGTCGAAGACTAGCGAA |
| D01390RB_F | ACTCGCTGGTAGTTACCACGTTCGGCCATCTAGGCCGGCTGTCATCTAGCGTTCTTCTCG |
| D01390RB_R | GCGGATAACAATTTCACACAGGAAACAGCCCTAAGGGACGCACAGGAAGCAAAGCGAATCA |
| Δros1 Complementation | |
| Cros1_F | GGTCCCATATGTCTGCATTCGATTCCACCTTGCTC |
| Cros1_R | TTTGCGGCCGCTTGCTACGTTCACGGTCGCTTTT |
| Cros1_KpnF | TAGTGGTACCTGGCTCTCGACATTCAGTCCTTG |
| Cros1_SbfR | GACTCCTGCAGGCGTCAGCGGACTGTTGAAATGTAA |
| Cros1_SbfF | GACTCCTGCAGGCTGCACGGCCGACGATCTTTT |
| Cros1_NotR | CATTGCGGCCGCTCACGGCACAGGTCTCGTCAACAC |
| TOPOros1_mF | CCAAGCTAGTGGTACTGCGCTCTCTCGCTCT |
| TOPOros1_mR | AGAGCGAGAGAGCGCAGTACCACTAGCTTGG |
| p123Bsu_F | ACATCTTCAGTCGCCTGAGGAGCGTCGTCGA |
| p123Bsu_R | TCGACGACGCTCCTCAGGCGACTGAAGATGT |
| ΔUMAG_02775 complementation | |
| P02775_F | GATCGGTACCAAATCTGCTTTCCAACTGGATTCG |
| P02775_R | GATCGGGCCCGATTTGCATGAACTGCTTCGTC |
| orf02775_F | GATCGGGCCCATGTCGTTGTCATCAACAAGCTA |
| orf02775_R | GATCGCGGCCGCTCAAATGATGCTGCGGATGTCCA |
| ΔUMAG_01390 complementation | |
| C01390_F | GATCGGTACCCACCAACTTAGCTCCTTCCGCTTT |
| C01390_R | GATCGGGCCCTCAGGCTTCGAGACCGTTGCTGCTT |
| *ros1* expression pattern | |
| ros1_qF | TGTCGAGATCGGTCCAGATGGAAT |
| ros1_qR | CTAGCAGGTCTGCCAAGTGTATCA |
| ppi-qF | ACATCGTCAAGGCTATCG |
| ppi-qR | AAAGAACACCGGACTTGG |
| Transcription factor genes expression pattern | |
| 02775_qF | ACAGCCAGTTCGCATCTTGA |
| 02775_qR | AGTTGCGATGAGCTGTCGAT |
| 01390_qF | AAGAGCAAAGCTGGCAGGAA |
| 01390_qR | TTGGGAGCATCCTCGCTTTT |
| Tup1_qF | TGCGCAGATCTTTGACACCA |
| Tup1_qR | AGCTCCCGTAGCCAAACATT |
| Rum1_qF | CAAAATGCGCAAGGCGATCA |
| Rum1_qR | CTTTGCCTTGGCAACCACTT |
| Ust1_qF | ACGTCAATCGGCTCCGATCT |
| Ust1_qR | TGCGAGCTCCGTTCACCAAT |
| Hgl1_qF | TCATCGAACAGGCACGCAAA |
| Hgl1_qR | AGATGGTTGGCCAAGACTGT |
| RNAseq validation | |
| 05550_qF | ACCAGTGGGATCTGCTCAAA |
| 05550_qR | ACCATGGTCGAAACCGTTCT |
| 04503_qF | GCCAAAGCGATTCTGCTCAA |
| 04503_qR | ACCAGCGTGAAGGTCAAAGT |
| 02212_qF | ACGTACATCGCACTTGAAGC |
| 02212_qR | TGCATCGGGATCAACGCAAA |
| 01070_qF | TCCATGACGCCTACTTTGCT |
| 01070_qR | TCGAACAAAGGCCAGGTGAA |
| Pks1_qF | TCGGTGATTAAGGCTGTGCT |
| Pks1_qR | AAAGATGAACCGGCGCAGAT |
| 04101_qF | ATTCCGTCTTGCCTTGGCAT |
| 04101_qR | AAGACTGTCGAGGACTGCAT |
| biz1_qF | GGATCAGCCAAATGATGGACAG |
| biz1_qR | TACTCTCGCATCTCTTCCACTC |
| rbf1_qF | AGTACGAGCTACGACGGATTC |
| rbf1_qR | GGGTAGGTGTTGGACACATTC |
| fox1_qF | TTCGTTCGCATCTCCAATCC |
| fox1_qR | TTGTCGCTGATCGCATTCTG |
| mig2-3_qF | AAGACGGACATCCTTTGCCT |
| mig2-3_qR | ACCTGCATACATGCACCTCT |
| 04096_qF | AGGCTACGATCGCCAACATT |
| 04096_qR | TTTGGCGCCATCAACTGGTA |
| dik1_qF | TGCAAATCCACGTCGCACTT |
| dik1_qR | AACGCGGTTCCACATGTTGC |
| 02473_qF | ATGGCTACAGCACCCTCAAA |
| 02473_qR | TGCCAGGCGTTCGGATATTT |
| 03046_qF | GACGCGGTTCTCAAAACTCT |
| 03046_qR | AGTCCAGTGCAAGTTGGTGA |
| Fungal biomass | |
| gapdh_qF | CTTCGGCATTGTTGAGGGTTTG |
| gapdh_qR | TCCTTGGCTGAGGGTCCGTC |
| Membrane marker | |
| MCsso1_NcoF | AATTCGATCCCATGGTGAGCAAG |
| MCsso1_HpaR | CAATGTTAACGCGCGCCCTTGATCTGCAACA |
| Psso1_kpnF | CATGGGTACCCTATGTCTCGGTCGCAGCTTTTC |
| Psso1_NcoR | CATGCCATGGGCGTTTCTTGGGTGCAAAATGTT |
| Nuclear marker | |
| Pnup_F | ATGTGGCTTACAAGCATTTCTGGA |
| Tnup_R | TGTGAAGAGATGGTCGCAGAGCTA |
| *ros1* ectopic expression | |
| crg-ros1LB_F | GTTCCATGGTCTGCATTCGATTCCACCTTGCTC |
| crg-ros1LB_R | GTTGAATTCGTTTGTCTGCCTTGGTCGAAGCTTT |
| crg-ros1RB_F | GTTCATATGATGGGTTCACACCAAGCTGCTACT |
| crg-ros1RB_R | GTTCCATGGTAGGCATTCCATCTGGACCGATCT |
| Pmig2-6_NdeF | CAATCATATGCTATTGCATCGTAGGCATTG |
| Pmig2-6_XmaR | CAATCCCGGGCTTGCAACACACTGATCGTGAAT |
| ChIP | |
| Ros1_XmaF | CTATCCCGGGATGGGTTCACACCAAGCTGCTA |
| Ros1_NotR | GTTGCGGCCGCACGGCACAGGTCTCGTCAACAC |
| Endros1_NcoF | ATGCCCATGGTTCCCAGTCAGCCGTCT |
| Endros1_PspR | GATTGGGCCCTAATAGTCGGGCACGTCGTAG |
| Expression in *E.coli* | |
| WOPR_NdeF | GTTACATATGGGTTCACACCAAGCTGCTACT |
| WOPR_XhoR | GTTACTCGAGCGACTCGCCAACGTACCTAGGCAT |
| EMSA probe generation | |
| WT-probe_F | CGCCAGAGACAAGTACTGTTC |
| WT-probe _R | ACAATAGCATACGCCTACGGA |
| ORF-probe_F | AGCCGCTCGCTGTCCATTTATT |
| ORF-probe _R  Probe-02854_F  Probe-02854_R  Probe-04040_F  Probe-04040_R  Probe-02538_F  Probe-02538_R  Probe-cmu1_F  Probe-cmu1_R  Probe-03046_F  Probe-03046_R  Probe-03138_F  Probe-03138_R  Probe-12258_F  Probe-12258_R  Probe-02775_F1  Probe-02775_R1  Probe-02775_F2  Probe-02775_R2 | GTTGGTAGGGTTCGAACCGTGAAT  GAGATACGAGACGAGTCAAGA  CAAGTTCCTCCAAAGAACCAC  CACTGCGGAGAGTGTCACAAA  AGAGAAGACAGGCATGGTCA  ATCGCGTCCTTTATGGTCAAAC  AGCCCTTGCCTGATTTGACGTA  CAGTTGCGTCGAGGATTCGT  GTTTCGCTCCAACTCGTCTT  AAGGTCACTCGACTGTAACTG  ACAGCCGCGTAGAATCTTG  GTAAGGTCGCCGCAACATC  AGGCCTGTTCAAAAGGTTGCT  CGAGGTTCCTCCGTTGTAGA  CCCTTACAGACCTCCAAAGA  TAGAAAGCCGGAGCAGTGAAC  CCTACCATCATAGCAAAACTC  CTTCGCTAATACTGAATGGCA  TCCAACAACTCTCGCGCTACT |
